# Beyond diagnostic categories: transdiagnostic psychological mechanisms associated with suicidal ideation among university students: a systematic review (2021–2025)

**DOI:** 10.3389/fpsyt.2026.1972940

**Published:** 2026-10-21

**Authors:** Diana Nathalie Onofre Ancco, Fabiola Mary Talavera-Mendoza, Maria Elena Rojas Zegarra

**Affiliations:** 1 Professional School of Psychology, Universidad Nacional de San Agustín de Arequipa, Arequipa, Peru; 2 Professional School of Education, Universidad Nacional de San Agustín de Arequipa, Arequipa, Peru

**Keywords:** emotion dysregulation, hopelessness, protective factors, rumination, suicidal ideation, systematic review, transdiagnostic processes, university students

## Abstract

**Introduction:**

Conventional diagnostic categories may not fully capture the psychological processes associated with suicidal ideation among university students. This systematic review synthesized recent evidence on candidate transdiagnostic psychological mechanisms, distinguishing ideation-specific findings from broader suicide-related outcomes.

**Methods:**

Scopus, Web of Science, PubMed, and PsycINFO via EBSCOhost were searched for peer-reviewed observational studies published between January 1, 2021, and December 18, 2025. Study selection followed PRISMA 2020, and methodological quality was assessed using design-specific Joanna Briggs Institute checklists. A narrative synthesis distinguished separately assessed suicidal ideation from complementary findings involving broad death/self-harm indicators, suicide-related cognitions, and composite suicide-risk scores. Classification depended on the outcome analyzed rather than instrument length.

**Results:**

Of 327 records identified, 68 studies were included; 58 were cross-sectional and 10 used longitudinal, prospective, or ecological momentary assessment designs. Findings were organized into cognitive–affective, emotional, behavioral, interpersonal–contextual, and protective domains. Ideation-specific analysis reported associations with hopelessness, repetitive negative thinking, perfectionistic concerns, depressive symptoms, perceived burdensomeness, and entrapment. Associations varied by outcome definition, statistical adjustment, and study design. Inverse or conditional associations involving psychological and interpersonal resources were reported in selected analysis. Complementary findings concerned broader suicide-related outcomes and were interpreted separately rather than as independent replication of ideation-specific associations.

**Discussion:**

The evidence identifies candidate psychological processes for further investigation, but predominantly cross-sectional designs and heterogeneous outcome measures limit causal and temporal interpretation. The proposed framework organizes observational findings rather than validating a causal model. Future prospective research should distinguish outcome constructs, address confounding, and test whether modifying candidate processes reduces suicidal ideation.

## 1 Introduction

Suicidal ideation is a major public health concern among university students, who may experience heightened psychological vulnerability in the context of academic demands, major life transitions, and social and emotional challenges. A systematic review and meta-analysis of young university students estimated a pooled lifetime prevalence of suicidal ideation of 27.1% and a past-year prevalence of 14.1%, although substantial variation was observed across individual studies (1). While university research has historically documented suicidal thoughts alongside clinical depression and general distress (2, 3), contemporary evidence underscores the need to determine how students appraise and respond to distress beyond formal psychiatric categories. This systematic review examines modifiable psychological processes associated with suicidal ideation in undergraduate and postgraduate populations.

Broader social circumstances, particularly the COVID-19 pandemic and its aftermath, may have intensified these vulnerabilities by increasing uncertainty, social isolation, academic disruption, and fear of failure (2). Exposure to adverse experiences, including victimization, family dysfunction, and chronic interpersonal stress, further correlates with elevated ideation and acute suicide risk (4–6). These findings demonstrate that suicidal thoughts emerge from an ecological matrix of individual, interpersonal, and environmental factors. However, conventional categorical diagnoses provide limited insight into how cognitive-affective vulnerabilities operate functionally within an individual student. A transdiagnostic perspective addresses this limitation by examining cognitive, emotional, behavioral, and interpersonal processes that cut across diagnostic boundaries and apply equally to subclinical student distress (7).

Integrative theoretical models of suicide provide conceptual foundations for conceptualizing these dynamic processes. The Interpersonal Theory of Suicide proposes that suicidal desire may emerge when perceived burdensomeness co-occurs with thwarted belongingness (8). Complementing this framework, the Three-Step Theory posits that psychological pain and hopelessness are central to the emergence of suicidal ideation (9). The Integrated Motivational–Volitional model situates defeat and entrapment at the core of the motivational phase of ideation, explicitly separating this phase from volitional factors that govern the transition to suicidal behavior (10). These models consistently indicate that suicidal ideation fluctuates dynamically and that static clinical markers cannot sufficiently capture immediate suicide risk (11).

Empirical research in university samples has evaluated several candidate transdiagnostic processes, including rumination, hopelessness, emotion dysregulation, maladaptive perfectionism, psychological inflexibility, negative affect, and impulsivity. Rumination prolongs negative affective states by entrenching recursive patterns of self-focused cognition. Emotion dysregulation involves deficits in identifying, modulating, or adaptively responding to emotional distress, frequently elevating vulnerability when coping resources are overwhelmed. Maladaptive perfectionism magnifies self-criticism, perceived deficiency, and catastrophic appraisals of academic setbacks. Impulsivity exhibits direct and indirect links to ideation (12), with intensive longitudinal research revealing that momentary state fluctuations in impulsivity covary with short-term surges in ideation rather than operating merely as a static trait (13). In parallel, maladaptive behaviors, such as experiential avoidance and social withdrawal, function as rigid coping responses that provide short-term distress reduction while consolidating persistent psychological vulnerability (7).

Despite the rapid accumulation of literature, the empirical evidence base remains conceptually and methodologically fragmented. In observational research, these processes represent candidate mechanisms: statistically, a correlation, regression coefficient, or indirect effect does not by itself establish a causal pathway. This distinction is critical for cross-sectional mediation models, which cannot establish whether a proposed mediator temporally precedes suicidal ideation (14). Additionally, primary studies frequently combine dedicated continuous ideation scales with single-item screening proxies (e.g., PHQ-9 item 9) or multidimensional composite suicide-risk indices that combine ideation with past behavior. Because brief screening items and composite scores do not capture the same construct as dedicated ideation assessments (15), unstratified syntheses risk obscuring true effect profiles.

To date, the recent evidence published between 2021 and 2025 on transdiagnostic psychological mechanisms associated with suicidal ideation among university students has not been comprehensively synthesized within a unified framework. Addressing this gap may contribute to a more integrated understanding of suicidal ideation and support the development of assessment and prevention strategies that focus on modifiable psychological mechanisms rather than relying exclusively on diagnostic categories. Therefore, this systematic review aimed to synthesize and critically examine transdiagnostic psychological mechanisms associated with suicidal ideation among university students, distinguishing primary evidence from separately assessed ideation, including explicit single items from complementary evidence involving brief screening proxies, proximal suicide cognitions, suicidal rumination, and multidimensional composite suicide-risk measures. This distinction organizes the synthesis according to the outcome analyzed and does not imply psychometric equivalence among measurement formats.

## 2 Methods

### 2.1 Study design and reporting guidelines

This study was conducted as a systematic review with narrative synthesis to identify, evaluate, and synthesize empirical evidence on transdiagnostic psychological mechanisms associated with suicidal ideation among university students. A meta-analysis was not performed because substantial conceptual and methodological heterogeneity was anticipated and observed across the included studies in terms of study designs, participant characteristics, operationalizations of psychological mechanisms, and measurement instruments. In addition, the included investigations reported non-combinable statistical estimates, precluding valid quantitative data pooling.

The review was conducted and reported in accordance with the Preferred Reporting Items for Systematic Reviews and Meta-Analyses 2020 statement (PRISMA 2020) (16). The PRISMA 2020 checklist will be provided as **Supplementary Material**. The review protocol was not prospectively registered.

### 2.2 Review question and eligibility framework

The review question and eligibility framework were structured using the Population–Exposure–Comparator Outcome (PECO) framework, which was considered appropriate because the review examined associations between psychological exposures and suicidal ideation rather than the effects of interventions. The review question was: What transdiagnostic psychological mechanisms are associated with suicidal ideation among university students?

The PECO components were defined as follows:

Population: Undergraduate and postgraduate students enrolled in universities or other postsecondary higher education institutions providing tertiary-level education. Studies involving clinical, nonclinical, or mixed student samples were eligible because the review focused on transdiagnostic psychological mechanisms that may occur across diagnostic categories and levels of psychological distress rather than on mechanisms restricted to a specific disorder. Clinical status was therefore not considered an exclusion criterion when participants met the educational eligibility criteria and suicidal ideation was assessed according to the predefined outcome criteria. Studies that included both university students and participants from other educational levels were considered only when data for the university student subgroup were reported separately and could be independently extracted.
Exposure: Transdiagnostic psychological mechanisms, defined as cognitive, emotional, behavioral, or interpersonal processes that may occur across different mental health conditions and are not restricted to a single diagnostic category. These mechanisms included, but were not limited to, hopelessness, rumination, emotion dysregulation, maladaptive perfectionism, psychological inflexibility, intolerance of uncertainty, negative affect, self-criticism, psychological pain, entrapment, loneliness, and impulsivity.
Comparator: University students with lower levels or an absence of the psychological mechanism under investigation, when the original study included a comparison group or comparison condition. A formal comparator was not required for observational or correlational studies.
Outcome: The principal review question concerned suicidal ideation assessed separately. Primary findings included dedicated ideation scales or subscales, explicit ideation items, and momentary ideation assessments. Complementary findings concerned broad death/self-harm indicators, suicide cognitions, suicidal rumination, mixed ideation/protective-cognition scores, composite suicide-risk scores, or distinct behavioral-history and motivational analysis. Classification depended on the analyzed construct and score, not instrument length. Studies contributed to both syntheses when distinct analysis concerned primary and complementary outcomes. This classification describes the synthesis and was not a prospectively specified eligibility hierarchy.

Suicidal ideation was conceptually delineated along a continuum distinguishing passive from active thoughts (1, 4). Passive suicidal ideation denotes a general desire or wish to die, or thoughts that one would be better off dead (e.g., wishing not to wake up), without active planning, explicit intent, or consideration of lethal means (4). In contrast, active suicidal ideation involves explicit, purposeful thoughts of taking one’s own life, encompassing varying degrees of intent, formulation of suicide plans, or preparation for lethal action (1, 4). Single-item screening indicators (e.g., PHQ-9 item 9) typically capture broad passive death wishes or self-harm thoughts without differentiating active suicidal intent.

Studies were eligible when they quantitatively evaluated an association between at least one transdiagnostic psychological mechanism and an eligible suicidal outcome: separately assessed suicidal ideation, a death/self-harm screening indicator, a proximal suicide-related cognitive construct, or a multidimensional composite suicide-risk score. The absence of a separately analyzed ideation outcome did not by itself require exclusion when an eligible complementary outcome was reported. Studies focusing exclusively on completed suicide, mortality, or isolated nonsuicidal self-injury without an eligible suicidal outcome were excluded.

### 2.3 Information sources and search strategy

A systematic literature search was conducted in Scopus, Web of Science Core Collection, PubMed, and PsycINFO via EBSCOhost. The search covered articles published from January 1, 2021, through December 18, 2025, which was the date of the final search update. No language restrictions were applied. The searches were limited to peer-reviewed journal articles, and full-text records were retrieved through institutional electronic subscriptions and open-access repositories.

The search strategies were customized to the specific indexing architecture and query syntax of each database, combining controlled vocabulary and free-text keywords where applicable. Controlled vocabulary (Medical Subject Headings [MeSH]) combined with title/abstract free-text terms was implemented exclusively in PubMed. In contrast, in PsycINFO via EBSCOhost, Scopus, and the Web of Science Core Collection, searches were executed exclusively through comprehensive free-text keyword strings without formal thesaurus restrictions (specifically utilizing the all-text field tag TX in PsycINFO via EBSCOhost, TITLE-ABS-KEY in Scopus, and topic terms TS in Web of Science) to maximize sensitivity and capture emerging transdiagnostic terminology across fields. The terms included “university students,” “college students,” “higher education students,” “suicidal ideation,” “suicidal thoughts,” “suicidality,” “emotion dysregulation,” “rumination,” “hopelessness,” “intolerance of uncertainty,” “perfectionism,” “psychological inflexibility,” “impulsivity,” and “negative affect”. Boolean operators (AND and OR) and field-specific tags were adapted to the indexing system and syntax of each information source.

All three authors participated in the development, review, and verification of the search strategies. The final searches retrieved 327 records: 86 from Scopus, 101 from Web of Science, 73 from PubMed, and 67 from EBSCOhost. No supplementary searches of reference lists, citation indexes, gray literature sources, or other information sources were conducted. The complete search strategies and the number of records retrieved from each source are presented in **Table 1**.

**Table 1:** Search strategies and records retrieved from each information source.

Information source	Search strategy	Records
Scopus	TITLE-ABS-KEY ((“university students” OR “college students” OR “higher education students”) AND (“suicidal ideation” OR “suicidal thoughts” OR suicidality) AND (“emotional dysregulation” OR rumination OR hopelessness OR “intolerance of uncertainty” OR perfectionism OR “psychological inflexibility” OR impulsivity OR “negative affect”))	86
Web of Science	TS=((“university students” OR “college students” OR “higher education students”) AND (“suicidal ideation” OR suicidality) AND (“emotional dysregulation” OR rumination OR hopelessness OR “intolerance of uncertainty” OR perfectionism OR “psychological inflexibility” OR impulsivity OR “negative affect”))	101
PubMed	((“Students”[MeSH] OR “college students”[Title/Abstract] OR “university students”[Title/Abstract]) AND (“Suicidal Ideation”[MeSH] OR “suicidal ideation”[Title/Abstract]) AND (“emotional dysregulation”[Title/Abstract] OR rumination[Title/Abstract] OR hopelessness[Title/Abstract] OR “intolerance of uncertainty”[Title/Abstract] OR perfectionism[Title/Abstract] OR “psychological inflexibility”[Title/Abstract] OR impulsivity[Title/Abstract] OR “negative affect”[Title/Abstract]))	73
PsycINFO	TX ((“university students” OR “college students”) AND “suicidal ideation” AND (“emotional dysregulation” OR rumination OR hopelessness OR “intolerance of uncertainty” OR perfectionism OR “psychological inflexibility” OR impulsivity))	67

The exposure search block comprised a predefined set of commonly investigated transdiagnostic psychological mechanisms and was not intended to constitute an exhaustive enumeration of all potentially relevant mechanisms. During screening and data extraction, additional cognitive-affective, emotional, behavioral, interpersonal, contextual, and protective mechanisms were retained when they were identified within records retrieved by the predefined search strategy and otherwise met the eligibility criteria. Accordingly, mechanisms not explicitly represented in the search terms are interpreted as findings identified within the retrieved literature rather than as the result of an exhaustive mechanism-specific search.

### 2.4 Eligibility criteria

Studies were selected according to predefined eligibility criteria aligned with the review question and the PECO framework.

#### 2.4.1 Inclusion criteria

- They were peer-reviewed empirical articles published in scientific journals between January 1, 2021, and 18 December, 2025.
- They employed a quantitative observational design, including analytical cross-sectional, longitudinal, prospective, or ecological momentary assessment designs.
- Their participants were undergraduate or postgraduate students enrolled in universities or other postsecondary higher education institutions providing tertiary-level education.
- When studies included participants from different educational levels or other populations, data corresponding to the eligible higher education student subgroup were reported separately and could be independently extracted.
- They examined at least one cognitive, affective, emotional, behavioral, interpersonal, contextual, or protective transdiagnostic psychological mechanism.
- They quantitatively evaluated suicidal ideation, a death/self-harm screening indicator, a proximal suicide-related cognitive construct (e.g., suicide cognitions or suicidal rumination), or a multidimensional composite suicide-risk score. Findings were subsequently organized for reporting according to the outcome analyzed, with broader outcomes interpreted separately from dedicated ideation assessments.

#### 2.4.2 Exclusion criteria

- They were systematic reviews, scoping reviews, narrative reviews, meta-analysis, qualitative studies, theoretical papers, editorials, letters, commentaries, protocols, or case reports.
- They were theses, dissertations, conference proceedings, conference abstracts, book chapters, preprints, or other forms of gray literature.
- Their participants were not undergraduate or postgraduate students enrolled in higher education institutions.
- They combined university students with participants from other populations without reporting separately extractable data for the university subgroup.
- They did not examine a transdiagnostic psychological mechanism in relation to any eligible suicidal outcome. The use of a non-disaggregated composite suicide-risk score alone was not an exclusion criterion.
- They focused exclusively on completed suicide, suicide mortality, isolated nonsuicidal self-injury, or historical suicide attempts without quantitatively evaluating suicidal ideation, death-related screening proxies, proximal suicide cognitions, or a validated multidimensional composite suicide-risk score capturing thoughts alongside behavior.
- They did not report sufficient empirical information or effect sizes to establish an association between the psychological mechanism under investigation and the suicidal outcome.
- They did not provide sufficient information for eligibility assessment, data extraction, or methodological quality appraisal.

### 2.5 Study selection

All records retrieved from the four information sources were imported into Microsoft Excel. Duplicate records were identified and removed before the screening process. Of the 327 records initially retrieved, 143 duplicates were removed, leaving 184 unique records for title and abstract screening.

Two reviewers independently screened the titles and abstracts of all identified records against the predefined eligibility criteria. At this stage, 96 records were excluded: 59 because they did not address the thematic focus of the review, 17 because the study population did not consist of university students, and 20 because they did not meet the methodological eligibility criteria. Consequently, 88 reports were sought for retrieval, and the full texts of all 88 reports were successfully obtained.

The same two reviewers independently assessed the 88 full-text articles for eligibility. Any uncertainty or disagreement regarding study inclusion was referred to a third reviewer, who reviewed the article and participated in the final decision. Disagreements were resolved through discussion and consensus among the reviewers.

Following the full-text assessment, 20 reports were excluded: 14 did not meet the thematic or outcome-related eligibility criteria, three did not meet the methodological eligibility criteria, one due to a mixed educational population without separately extractable higher-education results (Frey et al.), one lacked an eligible quantitative statistical association between mechanism and suicidal outcome, relying exclusively on descriptive opinion endorsements regarding causes of suicide (Alwan et al.), and one due to a non-eligible outcome, evaluating psychological suffering and hopelessness without a distinct suicidal outcome (Antúnez et al.). A total of 68 studies met all eligibility criteria and were included in the systematic review and narrative synthesis. The complete study selection process is presented in the PRISMA 2020 flow diagram (**Figure 1**).

**Figure 1:**
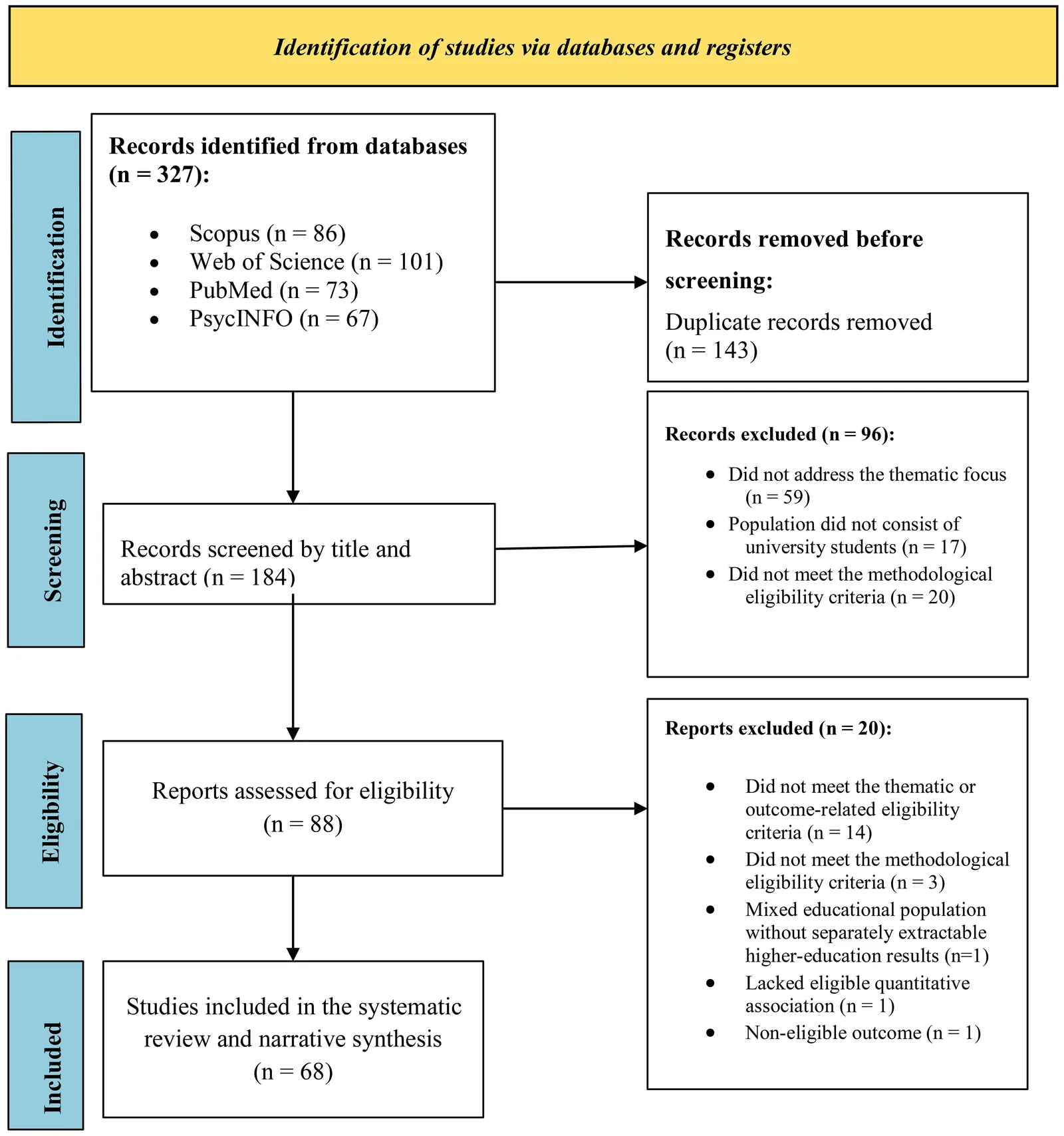
PRISMA 2020 flow diagram of the study identification, screening, eligibility assessment, and inclusion process.

### 2.6 Data extraction

Data were extracted using a standardized Microsoft Excel matrix developed for systematic reviews. The extraction template had been piloted with an initial subset of studies before its application to the complete sample to assess the clarity, relevance, and consistency of the predefined data fields.

One reviewer performed the initial extraction, organization, and condensation of the information from the included studies. A second reviewer verified the extracted data against the full-text articles to identify omissions, inconsistencies, or transcription errors. Any uncertainties or discrepancies identified during extraction and verification were referred to a third reviewer and resolved through discussion and consensus.

The following information was extracted from each study: authors, year of publication, country or geographical context, study design, participant characteristics and sample size, measurement instruments, transdiagnostic psychological mechanism examined, assessment of suicidal ideation, and principal findings. In addition, key statistical estimates (e.g., correlation coefficients, odds ratios, regression estimates, and statistical significance) were extracted to determine the direction, magnitude, and consistency of the reported associations.

Outcome extraction distinguished the instrument, analyzed item or subscale, scoring method, recall period, and target construct. Each study was categorized according to its extracted outcome metrics into one of three mutually exclusive groups: (a) primary evidence only, (b) complementary evidence only, or (c) both syntheses. Studies were classified under both syntheses when the primary report quantitatively evaluated both a dedicated suicidal ideation outcome (e.g., a specific ideation subscale or explicit ideation item) and a distinct complementary outcome (e.g., a composite risk total, broad depression screening proxy, or behavioral history/attempt classification) within separate analytical models. Studies contributing to both syntheses were counted once in the overall study total (N = 68) and independently in each analytical synthesis to which they contributed. **Table 2** details the study-level classification and the specific outcomes extracted for each investigation.

**Table 2:** Characteristics of the studies included in the systematic review (n = 68).

Study	Country	Sample and design	Transdiagnostic mechanism(s)	Target construct & instrument	Specific SI outcome extracted & measure type	Main findings
Akbulut et al. (72)	Türkiye	n=617; cross-sectional	Maladaptive thinking, emotion dysregulation, loneliness	SCS-R, 16 items	Continuous suicide-cognitions score [Multi-item proximal suicide-cognitions scale; distinct from direct suicidal ideation frequency]	Maladaptive thinking was positively associated with suicidal cognitions, with serial indirect associations through emotion-regulation difficulties and loneliness.
Arango-Tobón et al. (12)	Colombia	n=228; cross-sectional	Depression, hopelessness, impulsivity	ISO-30	ISO-30 total composite score [Composite suicidality measure]	Depression, hopelessness and impulsivity were examined against the composite ISO-30 score. An indirect association was reported; shared hopelessness content limits interpretation.
Camacho Gutiérrez et al. (3)	Mexico	n=158; cross-sectional	Depression, hopelessness	Beck Scale for SI, 19 items	Continuous SI total score [Multi-item SI scale; primary study findings interpreted with caution due to minor internal reporting discrepancies in subgroup counts].	Depression/hopelessness and SI were examined; inconsistencies in subgroup totals and correlation statistics limit interpretation of significance.
Cao et al. (4)	China	University subsample n=5,700; cross-sectional	Hopelessness, interpersonal relationships, polyvictimization	BSS	SI presence based on active/passive questions [Brief SI measure; not full BSS total]	Multiple forms of bullying were associated with greater SI in a dose-response pattern; hopelessness partly mediated these associations.
Chen et al. (42)	China	n=2,717; cross-sectional	Affective problems, hopelessness, bullying victimization	MINI-C, four questions	Reported score 0–17 [Structured interview ideation/suicidality module; continuous severity score with non-disaggregated item thresholds]	Bullying victimization was associated with SI; psychiatric prodromal experiences, rather than general affective problems, showed significant mediation.
Chesin and Jeglic (58)	USA	n=763; cross-sectional	Hopelessness, mindfulness, self-compassion	BSS	SI in the past week [Multi-item SI scale]	Compassion moderated the hopelessness-SI association. Global mindfulness should not be described as uniformly protective.
Daneshmend et al. (45)	Canada	n=539; cross-sectional	Impulsivity, childhood trauma, cannabis-related problems	CIDI suicidality items	Lifetime and past-year suicidal ideation, and suicide attempts assessed separately via CIDI [Both: Primary SI module & Complementary attempt history]	Problematic cannabis use, impulsivity, and childhood trauma were associated with suicidal ideation and attempts.
Dat et al. (62)	Japan	n=322; cross-sectional	Self-esteem, social anxiety, hopelessness	PHQ-9 item 9	Death/self-harm thoughts [Single-item broad proxy; not necessarily active SI]	Lower self-esteem and greater social anxiety were associated with SI; hopelessness partially mediated these associations.
Diao et al. (56)	China	n=546; cross-sectional	Digital stress, emotion regulation, meaning in life	PANSI, 14 items	Total including reversed protective items [Mixed negative/protective ideation score]	Reporting discrepancies prevented a firm interpretation of the direction of the cognitive-reappraisal association with the mixed PANSI outcome. No conclusive protective association was assigned.
Díaz Torres et al. (52)	Colombia	n=213; cross-sectional	Hopelessness, family functioning, substance-use risk	Plutchik Suicide Risk Scale, 15 items	Global suicide-risk score/status [Composite risk measure]	Composite suicide risk was associated with hopelessness, family dysfunction, sex and substance-use risk; findings should not be reported as isolated SI prevalence.
Drake and Sears (19)	Canada	n=284; cross-sectional	Hopelessness, humor styles	HDSQ suicidality subscale	Suicidality subscale score [Specific multi-item SI scale]	Hopelessness and self-defeating humor were positively associated with SI.
Etherson et al. (30)	United Kingdom	University sample n=240; three-wave longitudinal	Perfectionism, mattering, anti-mattering, acceptance of the past	ASIQ	Continuous SI total score [Specific multi-item SI scale]	Socially prescribed perfectionism prospectively predicted SI through anti-mattering and difficulty accepting the past.
Feng et al. (61)	China	n=1,011; cross-sectional	Rumination, depression, anxiety; physical activity	SI measure reported by authors	Continuous/ordinal SI score [Specific multi-item SI scale]	Lower physical activity was associated with greater rumination and depression, which formed an indirect pathway to SI.
Fernández-García et al. (66)	Spain	n=1,287; cross-sectional	Academic perfectionism, psychological well-being	Single SI item	Death as a release, past year [Single-item passive-death proxy]	Academic perfectionism was positively associated with SI, whereas academic performance and psychological well-being were protective.
Gerner et al. (11)	USA	n=43; 10-day EMA	Interpersonal hopelessness, burdensomeness, thwarted belongingness	Momentary suicidal desire items	Momentary suicidal desire rating [Ecological momentary assessment item]	Interpersonal hopelessness dimensions predicted momentary suicidal desire and were more stable than general hopelessness.
Gómez Tabares and Montalvo Peralta (49)	Colombia	n=422; cross-sectional	Family functioning, depression, experiential avoidance	ISO-30/SI subscale; Plutchik scale	Composite risk and specific ISO-30 SI subscale, separately [Both: Primary ISO-30 SI subscale & Complementary Plutchik/ISO-30 composite]	Poorer family functioning was associated with SI; depression mediated and experiential avoidance moderated the association.
Gnan et al. (67)	United Kingdom	n=385 LGBTQ+ students; prospective longitudinal	Negative beliefs, perseverative thinking, avoidance, self-compassion	SBQ-R	Total suicidality score [Composite history/frequency/future-risk measure]	Negative beliefs predicted subsequent composite suicidality. The proposed indirect effects through perseverative thinking/avoidance were nonsignificant; self-compassion was removed from the model.
Guo et al. (60)	China	n=783; cross-sectional	Failure mindset, meaning in life, spiritual coping	BSS	Continuous SI total score [Specific multi-item SI scale]	Failure mindset was positively associated with SI; meaning in life and spiritual coping showed protective indirect associations.
He et al. (59)	China	n=6,854; cross-sectional	Rumination, resilience, borderline personality traits	Separate lifetime SI and attempt questions	Lifetime SI status and lifetime attempts, separately [Both: Primary single-item SI status & Complementary attempt history]	Borderline traits were associated with ideation/attempt risk; resilience showed a protective association and rumination an adverse indirect association.
Hirsch et al. (5)	USA	n=338; cross-sectional	Hopelessness, self-compassion, negative life events	SBQ-R	SBQ-R total composite score [Composite suicide risk measure]	Negative life events and hopelessness were associated with suicide risk; self-compassion showed a protective moderating association.
Hovey et al. (18)	USA	n=279; cross-sectional	Perceived burdensomeness, coping competence, hopelessness	ASIQ	SI during the past month [Multi-item SI scale]	Perceived burdensomeness and hopelessness were positively associated with SI, whereas coping competence was protective.
Huang et al. (25)	China	n=831; cross-sectional	Rumination, entrapment, mindfulness	Self-rating Idea of Suicide Scale	SI score after excluding five lie-detection items [Multi-item measure]	Rumination was positively associated with SI; entrapment mediated the association and mindfulness attenuated it.
Iweama et al. (70)	Nigeria	n=625; cross-sectional	Depression, hopelessness, burdensomeness, belongingness	SBQ-R	SBQ-R total composite score [Composite suicidal behavior measure]	SI was associated with depression, hopelessness, and particularly perceived burdensomeness.
Kelly et al. (32)	USA	n=265; cross-sectional	Psychological pain, hopelessness, interpersonal needs, meaning	C-SSRS	SI severity subscale score (passive/active) [Specific multi-item SI scale]	Psychological pain was associated with passive and active SI; hopelessness and the pain–meaning discrepancy were especially relevant to active SI.
Kiaei and Kachooei (27)	Iran	n=300; cross-sectional	Perfectionism, self-criticism	BSS	Continuous SI total score [Specific multi-item SI scale]	Self-oriented and socially prescribed perfectionism were positively associated with SI, partly through self-criticism.
Langer et al. (47)	Chile	n=1,355; cross-sectional	Experiential avoidance, stress, psychopathology	SITBI	Suicidal ideation subscale/presence [Structured interview module]	Previous NSSI and suicidal behavior were strongly associated with recent and prospective SI; experiential avoidance moderated some associations.
Laskaja and Rozanov (6)	Russia	n=441; cross-sectional	Family emotional communication and parenting styles	SBQ-R	Extreme composite-risk groups and self-estimated future-attempt probability [Outcome-selected risk comparison]	Extreme-risk groups differed in family communication. Group selection and scoring/statistical uncertainties limit interpretation and generalizability.
Layrón et al. (35)	Spain	n = 737; six-month longitudinal EMA	Affect, emotion regulation, interpersonal needs, purpose in life	EMA SI items	Repeated SI frequency and trajectories in selected SI subgroup [EMA items]	Depressive symptoms, negative affect, burdensomeness, and low belongingness were associated with greater SI; purpose and positive affect were protective.
Lew et al. (71)	China	n=11,407; cross-sectional	Hopelessness, psychological pain, self-esteem, support, purpose	SBQ-R	SBQ-R total composite score [Composite suicidal behavior measure]	Religious affiliation showed differential associations with suicidal ideation and risk after adjustment for psychological and social factors.
Lew et al. (77)	China	n=11,806; cross-sectional	Depression, psychological pain, hopelessness, support, self-esteem, purpose	SBQ-R	SBQ-R total composite score [Composite suicidal behavior measure]	Depression and psychological pain were associated with suicide-attempt risk, whereas older age showed a protective association.
Littleton et al. (53)	USA	n=362 students with NSSI; cross-sectional	Burdensomeness, belongingness, loneliness, hopelessness, impulsivity	ISAS and suicide-history items	Self-reported SI history status [Single-item proxy]	Students with NSSI plus SI/attempts reported greater burdensomeness and loneliness and lower belongingness than comparison groups.
Liu et al. (24)	China	n=6,834; cross-sectional	Rumination, perceived social support, childhood sexual abuse	SCL-90 suicide item	Single-item death/suicide thought proxy [Complementary screening proxy]	Childhood sexual abuse was associated with SI; rumination mediated and social support attenuated the association.
Liu et al. (23)	China	n=674; two-wave longitudinal	Perfectionism, event-related rumination, depression	HDSQ suicidality subscale	Suicidality subscale score [Specific multi-item SI scale]	Perfectionism was prospectively associated with SI indirectly through rumination and depression; depression showed the proximal association.
Liu et al. (17)	China	n=1,191; cross-sectional	Rumination, hopelessness, gratitude	BSS	Continuous SI total score [Specific multi-item SI scale]	Hopelessness partially mediated the rumination–SI association; gratitude attenuated the hopelessness–SI association.
Lindquist et al. (64)	USA	n=1,816; cross-sectional	Fear of negative evaluation, negative urgency, perseverance	DSM-5 cross-cutting SI item	Single-item SI frequency/presence [Single-item proxy]	Fear of negative evaluation was associated with SI, particularly at higher negative urgency and lower perseverance.
Lucas and Chang (31)	USA	n=283; cross-sectional	Perfectionism, dark personality traits, negative affect	Frequency of SI Inventory	Continuous SI frequency score [Specific multi-item SI scale]	Psychopathy and parental criticism were significant correlates of SI after accounting for broader psychological maladjustment.
Mandel et al. (26)	USA	n=55; EMA	Rumination, hopelessness, psychological pain, attentional fixation	C-SSRS/momentar y passive SI	Momentary WTD and WTL, separately; active SI excluded from analysis [EMA ratings]	Rumination was examined in relation to momentary wish to die and wish to live. Active SI was excluded; adjusted and unadjusted findings should be distinguished.
Marraccini et al. (50)	USA	n=626; cross-sectional latent-class analysis	Depression, hopelessness, belongingness, executive dysfunction	SITBI/DSI-SS	SI/plans/attempts/NSSI profiles; recent SI/urges, separately [Both: Primary DSI-SS SI subscale & Complementary latent profiles].	Three self-injurious-thought/behavior profiles were identified; higher-risk profiles showed greater depression, hopelessness, and cognitive dysfunction.
Martinez-Fierro et al. (43)	Mexico	n=433 nursing students; cross-sectional	Hopelessness, depression, impulsivity, support, substance use	BSS	Reported suicidal-behavior classification [Composite ideation/attempt status; cutoff criteria operationalized by primary authors as presence of any suicidal desire or intent]	Hopelessness and depression were the most robust psychological correlates of suicidal behavior in the adjusted model.
McHugh et al. (38)	Ireland	n=1,829; cross-sectional	Depression, impulsivity, substance-use problems	SITBI/WMH- ICS items	Lifetime SI, plans, attempts and self-injury, separately [Both: Primary SI module & Complementary attempt/NSSI history]	Depression showed the strongest association with SI; impulsivity partially mediated associations involving depression and substance use.
Mills et al. (68)	Australia	n=302 doctoral students; cross-sectional	Loneliness, impostor phenomenon, perfectionism, supervisory relationship	SBQ-R	SBQ-R total composite score [Composite suicidal behavior measure]	Composite suicide risk was associated with loneliness, impostor feelings, perfectionism, prior mental-health problems, and disability.
Morshidi et al. (57)	Malaysia, Singapore and Australia	n=641; cross-sectional	Academic burnout, psychological pain, hopelessness	Suicide Ideation Scale, 10 items	Passive/active-ideation intensity [Multi-item SI scale]	Educational expectations were indirectly associated with SI through psychological pain, hopelessness, and academic burnout.
Moscardini et al. (33)	Multinatio nal	n=195; cross-sectional	Entrapment, meaning in life, reasons for living	BSSI	Continuous SI total score [Specific multi-item SI scale]	Entrapment was positively associated with SI, whereas meaning in life and reasons for living were protective.
Muneeb and Hassan (20)	Pakistan	n=400; cross-sectional	Psychological strain, rumination, depression	Suicide Ideation Scale, 10 items	SI intensity in the past week [Multi-item SI scale]	Psychological strain was associated with SI; depression and rumination contributed indirect pathways.
Oskouei et al. (21)	Iran	n=505 medical students; cross-sectional SEM	Perfectionism, repetitive negative thinking, loneliness	BSSI, 19 items	Continuous SI score; prevalence requires threshold clarification [Multi-item SI scale]	Perfectionism was indirectly associated with SI through repetitive negative thinking and loneliness; the model explained 30% of SI variance.
Pan et al. (46)	China	n=10,754; cross-sectional	Obsessive-compulsive symptoms, impulsivity	SBQ-R	SBQ-R total composite score [Composite suicidal behavior measure]	Obsessive-compulsive symptoms were associated with composite suicide risk, with impulsivity acting as a mediator.
Peng et al. (75)	China	n=592; cross-sectional	Family functioning, depression, experiential avoidance/acceptance	PANSI, 14 items	Total with reversed protective items [Mixed ideation/protective-cognition measure]	Poorer family functioning was associated with greater SI through depression; acceptance/experiential avoidance moderated the pathway.
Peng and Ren (51)	China	n≈1,400; cross-sectional network analysis	Depressive symptoms, burdensomeness, thwarted belongingness	BDI suicide item	Suicide item score (0–3 severity scale) [Single-item depression proxy]	SI was connected to depressive and interpersonal-need symptoms, with gender differences in central pathways.
Pérez et al. (63)	Spain	n=225; longitudinal with weekly EMA	Perceived burdensomeness (PB), thwarted belongingness (TB), hopelessness, depressive symptoms	PHQ-9 items 9	Complementary outcome [Complementary: EMA longitudinal PHQ-9 item 9 proxy]	In prospective path modeling evaluating follow-up PHQ-9 item 9 scores (63), the direct path from thwarted belongingness was nonsignificant (β = .149), whereas its contribution operated via hopelessness and a significant multiplicative interaction (PB × TB; β = .381).
Rodríguez et al. (78)	Spain	n=718; longitudinal/EMA	Depressive symptoms, purpose in life	PHQ-9	Item 9 score (death/self-harm thoughts) [Single-item depression proxy]	Purpose in life mediated the prospective association between depressive symptoms and SI frequency.
Rojano García and Altamirano López (44)	Ecuador	n=196; cross-sectional	Impulsivity	BSS	BSS categorical risk levels and raw score [Ordinal multi-item SI measure; primary report provided limited psychometric specification of interval thresholds]	Impulsivity showed a small positive association with SI; SI did not differ significantly by sex.
Shi et al. (69)	China	n=3,871; prospective longitudinal	Hopelessness, impulsivity, family conflict, reasons for living	SBQ-R	Three-wave composite suicide-risk trajectories [Composite suicide-risk measure; not isolated SI trajectories]	Two trajectories emerged: low-decreasing and high-increasing. Self-injury, hopelessness, emotional abuse, and depression predicted the high-risk trajectory, while reasons for living were protective. Impulsivity and family conflict lost significance after adjustment.
Sun et al. (41)	China	n=1,798; cross-sectional	Negative future disposition, somatic anxiety, general distress, positive affect	Multidimensiona l Suicide Inventory-28	SI over the past 2–3 weeks [Multi-item SI subscale]	Negative future disposition showed indirect associations with suicidal ideation through specific affective components, including somatic anxiety and general psychological distress, rather than operating through an undifferentiated depressive composite.
Tolegen et al. (54)	Kazakhstan	n=528; cross-sectional	Family conflict, perfectionism, anxiety, depression, stress tolerance	SBQ-14	SBQ-14 total score [Composite suicidal behavior measure]	Suicide risk was positively related to family conflict, perfectionism, anxiety, and depression and negatively related to stress tolerance and family cohesion.
Türk et al. (73)	Türkiye	n=470; cross-sectional	Psychological strain, suicidal rumination, depression, resilient coping, support	Suicidal Rumination Scale	Continuous suicidal rumination score [Specific multi-item scale]	Suicidal rumination was associated with psychological strain and depression; resilient coping and perceived support showed protective roles.
Vidal et al. (34)	Spain	n=524 (analytic sample; 650 recruited); cross-sectional	Defeat, entrapment, rumination	Risk Indicator Questionnaire (RIQ-13)	Global suicide risk score (13-item total score) [Composite suicide risk measure]	Defeat was indirectly associated with composite suicide risk through rumination and entrapment.
Vidal-Arenas et al. (65)	Four-country sample	n=3,482; cross-sectional	Neuroticism, rumination, depressive mood	DSM-5 self-harm/suicide screening item	Single-item death/self-harm thought proxy [Complementary screening indicator]	Rumination and depressive mood were positively associated with SI, whereas emotional stability was protective.
Wang et al. (48)	China	n=1,254; cross-sectional	Rumination, experiential avoidance, depression, childhood emotional abuse	SI and NSSI items	Explicit single-item suicidal ideation frequency [Primary single-item SI measure]	Childhood emotional abuse was associated with SI and NSSI through rumination, experiential avoidance, and depression.
Wei et al. (37)	China	n=1,895; longitudinal	Impostor phenomenon, depression, perceived social support	HDSQ suicidality subscale	Suicidality subscale score [Specific multi-item SI scale]	Impostor feelings were associated with greater depression and SI; family and peer support attenuated these associations.
Wu et al. (55)	China	n=1,458; cross-sectional	Rumination, regulatory emotional self-efficacy, childhood maltreatment	SBQ-R	SBQ-R total composite score [Composite suicidal behavior measure]	Childhood maltreatment was associated with suicidal behavior through rumination; regulatory emotional self-efficacy moderated the pathway.
Yamaguchi et al. (36)	Japan	n=370; cross-sectional	Emotional vulnerability, depressive symptoms	Short-form SI Scale	Continuous SI score [Specific multi-item SI scale]	Depressive symptoms were positively associated with SI; emotional vulnerability alone was nonsignificant but interacted with depression.
Yang et al. (74)	China	n=505; cross-sectional network analysis	Anxiety and depressive symptoms	PANSI, 14 items	Total score with reversed protective items [Complementary: Item-level network and mixed risk composite]	Anxiety and depression were connected with SI; death thoughts and hopelessness/suicidal thoughts were key bridge symptoms.
Yao et al. (22)	China	n=5,211; cross-sectional	Rumination, hope, negative life events	BSS	Continuous SI total score [Specific multi-item SI scale]	Negative life events were associated with suicidal ideation, with rumination and hope contributing to the observed association.
You et al. (28)	South Korea	n=420; cross-sectional	Maladaptive perfectionism, life stress, self-compassion	ASIQ	Continuous SI total score [Specific multi-item SI scale]	Maladaptive perfectionism was indirectly associated with suicidal ideation via life stress; self-compassion was inversely associated with suicidal ideation, and while a buffering interpretation was proposed, formal statistical moderation was not established in the reported analyses.
Zeifman et al. (76)	Canada	n=130; cross-sectional	Self-compassion, self-criticism, hopelessness	SBQ-R	SBQ-R total composite score [Composite suicidal behavior measure]	Self-compassion was negatively associated with self-reported suicidal behavior, whereas self-criticism was the strongest adverse correlate.
Zhang et al. (40)	Pakistan	n=920 premedical students; cross-sectional	Perfectionism, psychological capital, procrastination, test anxiety	SI Scale	Continuous/ordinal SI score [Specific multi-item SI scale]	Test anxiety and perfectionism were positively associated with SI; psychological capital showed a protective role.
Zhao et al. (29)	China	n=1,483; cross-sectional	Self- and socially prescribed perfectionism, appearance-based rejection sensitivity, loneliness	Single item adapted from the Frequency of Suicidal Ideation Inventory	Past-year suicidal ideation frequency, scored 0–4 [Ideation-specific single-item measure]	Socially prescribed perfectionism had a direct association with SI; loneliness and appearance-based rejection sensitivity formed significant indirect pathways.
Zhu and Lan (39)	China	n=663 students with disabilities; cross-sectional	Negative emotion, self-acceptance, hopelessness	SI Questionnaire	Continuous/ordinal SI score [Specific multi-item SI scale]	Negative emotion was positively and self-acceptance negatively associated with SI; hopelessness partly mediated or suppressed these associations.

Empirical studies included in the systematic review (N = 68 unique studies). Measurement formats and outcome constructs represent distinct descriptive dimensions. Overall, 34 studies contributed exclusively to primary ideation findings, 29 exclusively to complementary findings, and 5 studies contributed to both syntheses. Primary evidence (k = 39; 57.4%) informs the principal synthesis of separately assessed suicidal ideation (multi-item scales, EMA ratings, explicit single-item indicators, and structured interview modules). Complementary evidence (k = 34; 50.0%), audited separately, comprises broad death/self-harm screening indicators and passive thoughts (k = 12; 17.6%), multidimensional composite suicide-risk measures (k = 17; 25.0%), and proximal suicide-cognition, suicidal rumination, or mixed ideation/protective inventories (k = 5; 7.4%). Composite-score associations concern overall suicide risk rather than isolated ideation. SI, suicidal ideation; EMA, ecological momentary assessment; SEM, structural equation modeling; ASIQ, Adult Suicidal Ideation Questionnaire; BDI-II, Beck Depression Inventory-II; BSS/BSSI, Beck Scale for Suicidal Ideation; CIDI, Composite International Diagnostic Interview; C-SSRS, Columbia–Suicide Severity Rating Scale; DSI-SS, Depressive Symptom Inventory–Suicidality Subscale; HDSQ, Hopelessness Depression Symptom Questionnaire; ISAS, Inventory of Statements About Self-Injury; ISO-30, Inventory of Suicide Orientation; MINI-C, Mini International Neuropsychiatric Interview for Children and Adolescents; NSSI, non-suicidal self-injury; PANSI, Positive and Negative Suicide Ideation Inventory; PHQ-9, Patient Health Questionnaire-9; RIQ, Risk Indicator Questionnaire; SBQ-R/SBQ-14, Suicidal Behaviors Questionnaire–Revised; SCS-R, Suicidal Cognitions Scale–Revised; SITBI, Self-Injurious Thoughts and Behaviors Interview; WTD, wish to die; WTL, wish to live.

The extracted psychological mechanisms were subsequently organized into theoretically informed domains, including cognitive-affective, emotional, behavioral, interpersonal and contextual, and protective mechanisms. This categorization was used to structure the narrative synthesis while preserving the specific terminology and statistical findings reported in the original studies.

### 2.7 Methodological quality assessment

The methodological quality of the included studies was assessed using the Joanna Briggs Institute (JBI) critical appraisal checklist appropriate to each study design. The JBI Checklist for Analytical Cross-Sectional Studies was applied to analytical cross-sectional studies, whereas the JBI Checklist for Cohort Studies was applied to longitudinal, prospective, and ecological momentary assessment studies with repeated observations over time.

Two reviewers conducted the initial methodological quality assessment using a standardized appraisal matrix. Their ratings were subsequently compared, and uncertainties or disagreements were referred to a third reviewer. Final decisions were reached through discussion and consensus among the three reviewers. All three reviewers consulted the full-text articles when resolving appraisal questions and interpreting the evidence included in the narrative synthesis.

Each applicable JBI item was rated as “yes,” “no,” “unclear,” or “not applicable.” Affirmative responses were assigned one point, whereas negative or unclear responses were assigned zero points. Items rated as not applicable were excluded from the denominator. Because the JBI checklists contained different numbers of applicable items, the methodological quality score for each study was converted into a percentage of affirmative responses.

For descriptive stratification, studies meeting at least 80% of applicable criteria were classified as high quality, those meeting 50–79% as moderate quality, and those meeting less than 50% as low quality. These thresholds were defined by the review authors to facilitate qualitative comparison across study designs and were not used as eligibility criteria.

Among the 68 included studies, 50 (73.5%) were classified as having high methodological quality, 17 (25.0%) as having moderate methodological quality, and 1 (1.5%) as having low methodological quality. Methodological quality was considered when interpreting the consistency and robustness of the findings; however, no otherwise eligible study was excluded solely on the basis of its appraisal score.

### 2.8 Data synthesis

Given the conceptual, methodological, and statistical heterogeneity across the included studies, a meta-analysis was not considered appropriate. The studies differed substantially in their designs, participant characteristics, transdiagnostic mechanisms, suicidal ideation measures, analytical approaches, and reported effect estimates. Therefore, the findings were synthesized narratively.

The synthesis followed a structured and theoretically informed process. First, the extracted findings were tabulated and compared according to study characteristics, methodological design, psychological mechanism, suicidal ideation measure, direction of association, statistical significance, and methodological quality. Second, the transdiagnostic mechanisms were grouped into five analytical domains: cognitive-affective mechanisms, emotional mechanisms, behavioral mechanisms, interpersonal and contextual mechanisms, and protective mechanisms.

Within each domain, studies were synthesized according to the study designs represented, the direction and consistency of the reported associations with suicidal ideation, the statistical estimates reported by the original authors, and the methodological quality of the evidence. Findings from longitudinal, prospective, and ecological momentary assessment studies were distinguished from those derived from cross-sectional studies to avoid treating evidence of temporal relationships as equivalent to concurrent associations.

For transparency of the narrative synthesis, the evidence for each mechanism was summarized according to the number of contributing studies and the direction of the reported findings. Findings were categorized descriptively as positive/risk-related, negative/protective, mixed or conditional, or non-significant, according to the results reported by the original studies. These categories were used to describe patterns across studies and were not treated as pooled effect estimates. Where studies reported multiple models or conditional, mediating, or moderating effects, these findings were retained as mixed or conditional rather than reduced to a single direction of effect.

Positive, negative, mixed, and nonsignificant associations were retained in the synthesis to reduce selective emphasis on statistically significant findings. Mediation and moderation findings were reported as statistical associations proposed by the original studies and were not interpreted as evidence of causality unless temporal precedence was supported by the study design. Because of the predominance of observational evidence, the synthesis consistently used noncausal terminology, including “was associated with,” “was identified as a possible mediator,” and “the evidence suggests.”

Methodological quality was considered when interpreting the consistency and robustness of the findings but was not used to weight studies statistically or exclude otherwise eligible evidence. Particular interpretive emphasis was placed on mechanisms supported by multiple studies, findings replicated across different samples or geographical contexts, and associations observed in longitudinal or intensive repeated-measures designs.

## 3 Results

### 3.1 Study selection

The systematic search identified 327 records across the four information sources: 86 from Scopus, 101 from Web of Science, 73 from PubMed, and 67 from PsycINFO via EBSCOhost. After 143 duplicate records were removed, 184 unique records remained for title and abstract screening.

During the title and abstract screening stage, 96 records were excluded: 59 because they did not address the thematic focus of the review, 17 because the study population did not meet the eligibility criteria, and 20 because they did not meet the methodological or publication-type criteria. Consequently, 88 reports were sought for retrieval. The full texts of all 88 reports were successfully obtained and assessed for eligibility; therefore, no reports were classified as not retrieved.

Following the full-text assessment, 20 reports were excluded: 14 did not meet the thematic or outcome-related eligibility criteria, three did not meet the methodological eligibility criteria, one due to a mixed educational population without separately extractable higher-education results (Frey et al.), one lacked an eligible quantitative statistical association between mechanism and suicidal outcome, relying exclusively on descriptive opinion endorsements regarding causes of suicide (Alwan et al.), and one due to a non-eligible outcome, evaluating psychological suffering and hopelessness without a distinct suicidal outcome (Antúnez et al.). A total of 68 studies met all eligibility criteria and were included in the systematic review and narrative synthesis. The complete study selection process is presented in the PRISMA 2020 flow diagram (**Figure 1**).

### 3.2 Characteristics of the included studies

The 68 included studies were published between 2021 and 2025. Ten studies (14.7%) were published in 2021, 13 (19.1%) in 2022, 11 (16.2%) in 2023, 16 (23.5%) in 2024, and 18 (26.5%) in 2025. Thus, approximately half of the evidence base was published during the final two years of the review period, indicating increasing research attention to psychological mechanisms associated with suicidal ideation among university students.

The studies represented a geographically diverse evidence base. China was the most frequently represented country, accounting for 24 studies (35.3%), followed by the United States with 10 studies (14.7%) and Spain with five studies (7.4%). Additional studies were conducted in Australia, Brazil, Canada, Chile, Colombia, Ecuador, India, Iran, Ireland, Japan, Mexico, Nigeria, Pakistan, Russia, Kazakhstan, Malaysia, Singapore, South Korea, Türkiye and the United Kingdom. Several studies included participants from more than one country.

Most studies used analytical cross-sectional designs (n = 58; 85.3%). The remaining 10 studies (14.7%) used longitudinal, prospective, cohort, or ecological momentary assessment designs. Sample sizes ranged from 43 participants in an ecological momentary assessment study to 11,806 participants in a large multicenter survey. Most studies included undergraduate students, although some examined postgraduate, medical, nursing, doctoral, or other higher-education students. Several studies focused on specific university subgroups, including student athletes, students with disabilities, students reporting recent suicidal ideation, and students exposed to particular psychosocial stressors.

Considerable heterogeneity was observed in the psychological mechanisms examined and in the measures used to assess suicidal ideation. The mechanisms most frequently investigated included hopelessness, rumination, emotion-regulation difficulties, perfectionism, impulsivity, loneliness, perceived burdensomeness, thwarted belongingness, psychological pain, entrapment, experiential avoidance, self-compassion, resilience, meaning in life, and social support. Suicidal ideation was assessed using multi-item instruments, subscales of broader suicide-related measures, ecological momentary assessment items, and, in some studies, individual items from depression measures. Single-item assessments, including item 9 of the Patient Health Questionnaire-9, were retained only when the original study explicitly operationalized and analyzed the item as an indicator of suicidal ideation.

Measurement approaches were classified according to the specific construct analyzed rather than instrument length alone (N = 68 unique studies). Overall, 34 studies (50.0%) contributed exclusively to primary ideation findings, 29 studies (42.6%) contributed exclusively to complementary findings, and 5 studies (7.4%; Daneshmend et al., Gómez-Tabares & Montalvo-Peralta, He et al., Marraccini et al., McHugh et al.) contributed to both syntheses by reporting separate analysis for primary ideation and complementary outcomes (such as composite indices, broad proxies, or distinct behavioral-history profiles). Across these categories: (1) Primary evidence (k=39; 57.4% of the study base) comprised separately evaluated suicidal ideation assessed via dedicated multi-item continuous scales or subscales (k=31; e.g., BSS, ASIQ, C-SSRS, ISO-30 SI subscale), repeated-measures ecological momentary assessment (EMA) ratings of momentary suicidal desire (k=2; Gerner et al., Layrón et al.), explicit single-item ideation measures (k=3), or structured interview ideation modules (k=3). (2) Complementary evidence (k=34; 50.0% of the study base), audited separately to avoid false construct equivalence, was operationalized into: (a) broad death- or self-harm-related screening indicators and passive thoughts (k=12; 17.6%; e.g., PHQ-9 item 9, SCL-90 suicide item, DSM-5 proxy, including longitudinal EMA), (b) multidimensional composite suicide-risk measures combining thoughts with prior behavioral enactment (k=17; 25.0%; e.g., SBQ-R, SBQ-14, ISO-30 total composite, Plutchik Suicide Risk Scale, RIQ-13), and (c) proximal suicide-specific cognitions, suicidal rumination, or mixed ideation/protective scores (k=5; 7.4%; SCS-R, and mixed PANSI). To preserve construct validity, screening proxies and composite scores were interpreted strictly as broader indicators of suicide-related vulnerability rather than treated as psychometrically equivalent to dedicated suicidal ideation assessments, (d) distinct behavioral-history comparisons or latent-class attempt profiles (k = 4; lifetime attempt history, latent self-injury profiles, and separate attempt/self-injury models).

The main characteristics of the included studies, including; country, sample, study design, psychological mechanisms, suicidal ideation measures, and principal findings, are summarized in **Table 2**.

### 3.3 Methodological quality of the included studies

The methodological quality appraisal indicated that the overall evidence base was of generally high quality. Of the 68 included studies, 58 (85.3%) were cross-sectional and 10 (14.7%) employed longitudinal, prospective cohort, or ecological momentary assessment designs. Across the synthesized sample, 50 studies (73.5%) were classified as having high methodological quality, 17 (25.0%) as moderate quality, and one (1.5%) as low quality.

Most studies adequately described their eligibility criteria, participants, settings, psychological variables, and statistical analysis. The moderate-quality ratings primarily reflected incomplete reporting regarding the identification and management of potential confounding factors, measurement procedures, or follow-up strategies, as applicable to the study design. These limitations were considered when interpreting the consistency and robustness of the findings, particularly because most of the evidence was derived from cross-sectional studies.

No otherwise eligible study was excluded solely on the basis of its methodological quality. The complete item-level JBI appraisal for each included study is presented in **Supplementary Table S1**.

### 3.4 Transdiagnostic psychological mechanisms associated with suicidal ideation

This section synthesizes findings in which suicidal ideation was assessed separately from broader suicide-risk composites and related cognitive outcomes (**Figure 2**). To preserve construct validity and account for measurement variations across higher education research, the synthesis focuses primarily on candidate processes examined in relation to dedicated ideation assessments across five interconnected domains (**Table 3**): cognitive–affective processes, emotional processes, behavioral characteristics, interpersonal and contextual variables, and potentially protective resources. These domains organize the candidate mechanisms identified within primary studies; they do not imply that every variable represents a demonstrated or causal mechanism.

**Figure 2:**
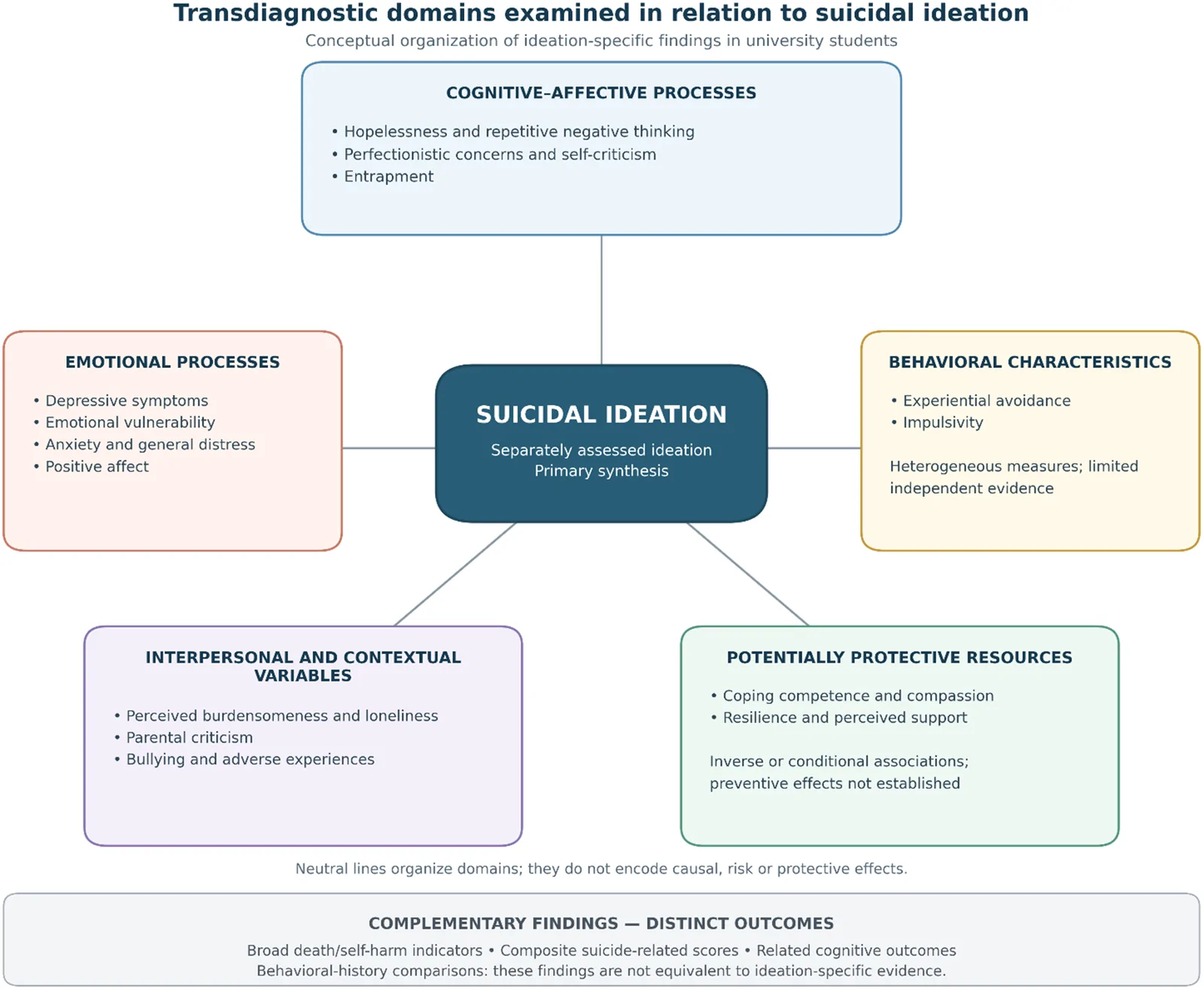
Conceptual organization of transdiagnostic domains examined in relation to suicidal ideation among university students. The central node represents separately assessed ideation. The five surrounding domains contain examples of examined variables; neutral connecting lines indicate their organization within the primary synthesis and do not encode significant, independent, causal, or uniformly protective effects. Associations varied by measure, sample, adjustment, and design, and included conditional, attenuated, and nonsignificant findings. The lower band identifies complementary outcomes that are not interchangeable with suicidal ideation. The figure does not represent a validated causal model, temporal sequence, or transition from ideation to suicidal behavior.

**Table 3:** Synthesis of transdiagnostic psychological mechanisms associated with suicidal ideation among university students.

Domain	Psychological mechanism	Total studies contributing (k)	Direction of reported findings	Contributing studies	Synthesized finding
Cognitive–affective	Hopelessness	k=25	Direct/bivariate risk: k=13 Indirect/path or profile association: k=11 Nonsignificant in final adjusted model: k=1	**Direct/bivariate risk:** Arango-Tobón et al. (12); Camacho Gutiérrez et al. (3); Chesin and Jeglic (58); Díaz Torres et al. (52); Drake and Sears (19); Gerner et al. (11); Hirsch et al. (5); Hovey et al. (18); Iweama et al. (70); Kelly et al. (32); Lew et al. (71); Lew et al. (77); Littleton et al. (53). **Indirect/path or profile association:** Cao et al. (4); Chen et al. (42); Dat et al. (62); Liu et al. (17); Mandel et al. (26); Marraccini et al. (50); Martinez-Fierro et al. (43); Morshidi et al. (57); Pérez et al. (63); Shi et al. (69); Zhu and Lan (39). **Nonsignificant in final adjusted model:** Zeifman et al. (76).	Hopelessness was associated with several suicide-related outcomes, but adjusted results were mixed. Indirect associations, profile differences and composite-risk findings are not equivalent to a demonstrated causal mediator.
Cognitive–affective	Rumination and repetitive negative thinking	k=14	Direct/bivariate association: k=4 Indirect/path association: k=8 Attenuated/direct null after adjustment: k=3 Different outcome (suicidal rumination): k=1 Composite risk outcome (RIQ-13): k=1	**Direct/bivariate association:** He et al. (59); Huang et al. (25); Liu et al. (17); Mandel et al. (26). **Indirect/path association:** Feng et al. (61); Liu et al. (24); Liu et al. (23); Muneeb and Hassan (20); Oskouei et al. (21); Yao et al. (22); Vidal-Arenas et al. (65); Wang et al. (48). **Attenuated/direct null after adjustment:** Liu et al. (23); Mandel et al. (26); Vidal-Arenas et al. (65). **Different outcome (suicidal rumination):** Türk et al. (73). **Composite risk outcome:** Vidal et al. (34).	Perseverative negative thinking and brooding correlated with suicidal outcomes, primarily functioning as statistical intermediaries or indirect path conduits in multivariable models; independent prospective associations were substantially attenuated when controlling for concurrent depressive affect.
Cognitive–affective	Perfectionism and self-criticism	k=12	Maladaptive/socially prescribed perfectionism (risk): k=11 Self-criticism correlate/indirect path: k=2 Self-oriented perfectionism (mixed/direct null): k=3	**Maladaptive/socially prescribed perfectionism (risk):** Etherson et al. (30); Fernández-García et al. (66); Kiaei and Kachooei (27); Liu et al. (23); Lucas and Chang (31); Mills et al. (68); Oskouei et al. (21); Tolegen et al. (54); You et al. (28); Zhang et al. (40); Zhao et al. (29). **Self-criticism correlate/indirect path:** Kiaei and Kachooei (27); Zeifman et al. (76). **Self-oriented perfectionism (mixed/direct null):** Etherson et al. (30); Kiaei and Kachooei (27); Zhao et al. (29).	Socially prescribed and maladaptive perfectionism, along with self-criticism, were consistently associated with suicidal ideation and composite suicide risk via anti-mattering, loneliness, and depression; self-oriented perfectionism showed inconsistent or null associations.
Cognitive–affective	Psychological pain	k=5	Direct/bivariate association: k=4 Indirect/mediator: k=1	**Direct/bivariate association:** Kelly et al. (32); Lew et al. (71); Lew et al. (77); Mandel et al. (26). **Indirect/mediator**: Morshidi et al. (57).	Elevated psychache correlated with both passive and active ideation as well as composite risk, functioning also as an indirect pathway connecting academic burnout to suicidal thoughts.
Cognitive–affective	Defeat and entrapment	k=3	Direct/indirect association: k=2 Composite risk pathway: k=1	**Direct/indirect association:** Huang et al. (25); Moscardini et al. (33). **Composite risk pathway:** Vidal et al. (34).	Internal and external entrapment consistently connected feelings of defeat and perseverative rumination with elevated suicidal ideation across dedicated continuous scales, complementary composite risk associations showed analogous indirect pathways.
Emotional	Emotion-regulation difficulties	k=4	Dedicated SI/momentary assessments: k=1 Suicide-cognitions outcome (not direct SI): k=1 Adaptive/regulatory association: k=1 Directionally uncertain/reporting discrepancies: k=1	**Dedicated SI/momentary assessments:** Layrón et al. (35). **Suicide-cognitions outcome:** Akbulut et al. (72). **Adaptive/regulatory association:** Wu et al. (55). **Directionally uncertain/reporting discrepancies:** Diao et al. (56).	In an intensive repeated-measures investigation utilizing standardized continuous ideation assessments, difficulties in identifying, modulating, and accepting emotional distress covaried with momentary suicidal ideation over time.
Emotional	Depressive symptoms	k=25	Direct/bivariate/node or profile association: k=17 Indirect/path association: k=8	**Direct/bivariate/node or profile association:** Arango-Tobón et al. (12); Camacho Gutiérrez et al. (3); Iweama et al. (70); Layrón et al. (35); Lew et al. (71); Lew et al. (77); Marraccini et al. (50); Martinez-Fierro et al. (43); McHugh et al. (38); Peng and Ren (51); Shi et al. (69); Gómez Tabares and Montalvo Peralta (49); Tolegen et al. (54); Türk et al. (73); Vidal-Arenas et al. (65); Yamaguchi et al. (36); Yang et al. (74). **Indirect/path association:** Chen et al. (42); Feng et al. (61); Liu et al. (23); Muneeb and Hassan (20); Peng et al. (75); Rodríguez et al. (78); Wei et al. (37); Wang et al. (48).	Depressive symptom severity acted as the most pervasive concurrent correlate and proximal mediator; however, shared item variance and lack of confounder adjustment in several primary studies warrant interpretive caution.
Emotional	Anxiety, stress, and psychological strain	k=12	Direct/indirect or descriptive association: k=11 Attenuated/nonsignificant adjusted affective path: k=1	**Direct/indirect or descriptive association:** Dat et al. (62); Diao et al. (56); Feng et al. (61); Langer et al. (47); Muneeb and Hassan (20); Sun et al. (41); Tolegen et al. (54); Türk et al. (73); Yang et al. (74); You et al. (28); Zhang et al. (40). **Attenuated/nonsignificant adjusted affective path:** Chen et al. (42).	Anxiety, stress and strain showed associations in several reports, but results were not uniformly independent after adjustment.
Emotional	Negative affect and emotional vulnerability	k=4	Negative-affect association: k=2 Conditional interaction: k=1 Indirect/suppression path: k=1	**Negative-affect association:** Layrón et al. (35); Lucas and Chang (31). **Conditional interaction:** Yamaguchi et al. (36). **Indirect/suppression path:** Zhu and Lan (39).	Negative affect was concurrently associated with suicidal ideation across cross-sectional and momentary assessments; emotional vulnerability operated primarily as a conditional risk factor, becoming salient when interacting with elevated depression or when mediated by hopelessness.
Behavioral	Impulsivity	k=10	Direct/bivariate association: k=2 Weak/modest bivariate correlation (limited threshold reporting): k=1 Indirect/moderator or group contrast: k=6 Nonsignificant after adjustment: k=1	**Direct/bivariate association:** Martinez-Fierro et al. (43); Shi et al. (69). **Weak/modest bivariate correlation (limited threshold reporting):** Rojano García and Altamirano López (44). **Indirect/moderator or group contrast:** Arango-Tobón et al. (12); Daneshmend et al. (45); McHugh et al. (38); Pan et al. (46); Lindquist et al. (64); Littleton et al. (53). **Nonsignificant after adjustment:** Shi et al. (69).	Impulsivity was associated with suicidal ideation and composite suicide risk, operating predominantly as a mediator or conditional moderator in pathways involving depression, OCD symptoms, and substance misuse; its independent direct effect was attenuated in one prospective multivariable model and showed small bivariate magnitude in another.
Behavioral	Experiential avoidance	k=5	Direct/conditional/path association: k=4 Proposed suicidality indirect path nonsignificant: k=1	**Direct/conditional/path association:** Langer et al. (47); Peng et al. (75); Gómez Tabares and Montalvo Peralta (49); Wang et al. (48). **Proposed suicidality indirect path nonsignificant:** Gnan et al. (67).	Experiential avoidance showed positive direct associations with suicidal ideation and composite suicide risk, while also moderating or mediating pathways connecting childhood adversity, family dysfunction, and depression to suicidal outcomes.
Behavioral	Nonsuicidal self-injury and previous suicidal behavior	k=9	Concurrent/history-related comparison: k=8 Prospective risk-trajectory association: k=1	**Concurrent/history-related comparison:** Chesin and Jeglic (58); Daneshmend et al. (45); Langer et al. (47); Lew et al. (71); Mandel et al. (26); Marraccini et al. (50); Wang et al. (48); Littleton et al. (53). **Prospective risk-trajectory association:** Shi et al. (69).	Histories of nonsuicidal self-injury or suicidal behavior were associated with ideation-related or composite-risk outcomes in concurrent comparisons. In Shi et al., these histories were prospectively associated with membership in persistent high composite suicide-risk trajectories. These findings do not establish a causal mechanism or trajectories of isolated suicidal ideation.
Behavioral	Substance-use problems	k=4	Direct/bivariate or indirect association: k=4 Attenuated after adjustment: k=2	**Direct/bivariate or indirect association:** Daneshmend et al. (45); Díaz Torres et al. (52); Martinez-Fierro et al. (43); McHugh et al. (38). **Attenuated after adjustment:** Martinez-Fierro et al. (43); McHugh et al. (38).	Problematic cannabis and alcohol use were associated with suicidal ideation and composite suicide risk, particularly when co-occurring with high impulsivity or trauma; however, independent direct associations were substantially attenuated after adjusting for depressive symptoms across two studies.
Interpersonal–contextual	Loneliness and social disconnection	k=5	Direct/group association: k=2 Indirect/path association: k=2 Indirect/serial path to suicide cognitions: k=1	**Direct/group association:** Mills et al. (68); Littleton et al. (53). **Indirect/path association to dedicated suicidal ideation:** Oskouei et al. (21); Zhao et al. (29). **Indirect/serial path to suicide cognitions (not direct SI):** Akbulut et al. (72).	Loneliness was directly associated with elevated suicidal ideation and composite suicide risk, while also emerging as a statistically significant indirect intermediary in cross-sectional path models involving maladaptive thinking, perfectionism, and appearance-based rejection sensitivity.
Interpersonal–contextual	Perceived burdensomeness and thwarted belongingness	k=8	PB direct/indirect/node association: k=8 TB direct/indirect association via hopelessness: k=5 TB independent direct main path null: k=1 PBxTB interaction: k=1	**PB direct/indirect/node association:** Gerner et al. (11); Hovey et al. (18); Iweama et al. (70); Kelly et al. (32); Marraccini et al. (50); Peng and Ren (51); Pérez et al. (63); Littleton et al. (53). **TB direct/indirect association via hopelessness:** Gerner et al. (11); Iweama et al. (70); Marraccini et al. (50); Peng and Ren (51). **TB prospective indirect path via hopelessness and interaction:** Pérez et al. (63). **PBxTB interaction:** Pérez et al. (63).	Findings for perceived burdensomeness and thwarted belongingness varied across outcomes and analyses. In prospective path modeling evaluating follow-up PHQ-9 item 9 scores (63), the direct path from thwarted belongingness was nonsignificant (β = .149), whereas its contribution operated via hopelessness and a significant multiplicative interaction (PB × TB; β = .381). These findings reflect broad death/self-harm thoughts and do not confirm an ideation-specific causal mechanism.
Interpersonal–contextual	Family dysfunction and conflict	k=6	Direct/indirect or group association: k=5 Exploratory factor/correlation: k=1 Attenuated after adjustment: k=1	**Direct/indirect or group association:** Díaz Torres et al. (52); Gómez Tabares and Montalvo Peralta (49); Laskaja and Rozanov (6); Peng et al. (75); Shi et al. (69). **Exploratory factor/correlation:** Tolegen et al. (54). **Attenuated after adjustment:** Shi et al. (69).	Family dysfunction, conflict, authoritarian climate, and low cohesion were recurrently associated with greater suicidal ideation and composite suicide risk; however, independent prospective direct associations were attenuated in one multivariable model after adjusting for depression and trauma.
Interpersonal–contextual	Childhood adversity and bullying	k=7	Direct/group/prospective association: k=3 Indirect/path association: k=4 Dose-response pattern: k=2	**Direct/group/prospective association:** Cao et al. (4); Daneshmend et al. (45); Shi et al. (69). **Indirect/path association:** Chen et al. (42); Liu et al. (24); Wu et al. (55); Wang et al. (48). **Dose-response pattern:** Cao et al. (4); Chen et al. (42).	Adversity and bullying were related to several suicide-related outcomes; dose-response and indirect pathways require their original measures and models.
Interpersonal–contextual	Academic and digital stressors	k=4	Direct/indirect/path association: k=4	**Direct/indirect/path association:** Diao et al. (56); Fernández-García et al. (66); Morshidi et al. (57); Zhang et al. (40).	Academic burnout, demanding performance expectations, and digital stress demonstrated indirect statistical associations with suicidal ideation and composite risk, modeled via intermediary paths involving cognitive-affective distress, emotional dysregulation, and psychological pain.
Protective	Self-compassion and mindfulness	k=5	Negative/conditional association: k=3 Proposed buffer not formally verified: k=1 Explicit-risk association; implicit outcome nonsignificant: k=1	**Negative/conditional association:** Chesin and Jeglic (58); Hirsch et al. (5); Huang et al. (25). **Proposed buffer not formally verified:** You et al. (28). **Explicit-risk association; implicit outcome nonsignificant:** Zeifman et al. (76).	Inverse or moderating associations were reported in selected analyses, but findings varied by construct, outcome measure, and statistical adjustment. A proposed buffering association was not formally established in one study. Self-compassion was inversely associated with self-reported composite suicide risk in another study, whereas its association with an implicit suicide task was nonsignificant; this difference in significance does not establish a difference between mechanisms.
Protective	Resilience and coping competence	k=5	Negative/conditional association: k=4 Exploratory factor/correlation: k=1	**Negative/conditional association:** He et al. (59); Hovey et al. (18); Türk et al. (73); Zhang et al. (40). **Exploratory factor/correlation:** Tolegen et al. (54).	Resilience/coping was examined against ideation, lifetime outcomes or suicidal rumination. Consistency of protective effects cannot be assumed across these measures.
Protective	Meaning, purpose, and reasons for living	k=8	Negative/conditional or trajectory association: k=6 Proposed prospective indirect association: k=1 Directionally uncertain/reporting discrepancies: k=1	**Negative/conditional or trajectory association:** Guo et al. (60); Kelly et al. (32); Layrón et al. (35); Lew et al. (71); Moscardini et al. (33); Shi et al. (69). **Proposed prospective indirect association:** Rodríguez et al. (78). **Directionally uncertain/reporting discrepancies:** Diao et al. (56).	Presence of meaning in life, purpose, and reasons for living exhibited consistent negative associations with suicidal ideation and composite suicide risk, acting as a cross-sectional mediator and prospectively differentiating lower-risk courses from chronic high-risk composite suicide-risk trajectories.
Protective	Gratitude, self-acceptance, and self-esteem	k=3	Negative/conditional association: k=3	**Negative/conditional association:** Dat et al. (62); Liu et al. (17); Zhu and Lan (39).	Distinct positive self-appraisal constructs were examined with SI or a broad proxy; they should not be treated as interchangeable protective mechanisms.
Protective	Perceived social support	k=4	Negative/conditional or adjusted association: k=4	**Negative/conditional or adjusted association:** Liu et al. (24); Martinez-Fierro et al. (43); Türk et al. (73); Wei et al. (37).	Support was examined in association with SI, broad suicide-related outcomes or suicidal rumination. Evidence is mainly concurrent or only partly prospective.

k indicates the number of unique empirical studies contributing to each transdiagnostic mechanism (n = 68 total primary studies reviewed). Because primary studies often tested multiple independent paths or serial models, the sum of directional subcategories may exceed k. Classifications as risk-related, protective, mediating, or moderating reflect the reported analytical models and do not demonstrate causality or temporal ordering. Detailed qualitative characteristics and individual study designs are presented in **Table 2**. SI, suicidal ideation; PB, perceived burdensomeness; TB, thwarted belongingness.

Bold text in the Domain column denotes the primary analytical categories of transdiagnostic mechanisms.

Across the primary evidence base, suicidal ideation was concurrently associated with maladaptive cognitive patterns, heightened emotional distress, behavioral disinhibition, and interpersonal thwartedness. Conversely, adaptive emotion regulation, meaning in life, resilience, and perceived social support consistently coincided with lower ideation severity. Given the predominance of analytical cross-sectional designs (85.3%), these results represent statistical associations rather than verified causal pathways. Findings from prospective cohort and ecological momentary assessment designs are distinguished to highlight preliminary evidence of temporal ordering.

#### 3.4.1 Cognitive–affective mechanisms

Cognitive-affective mechanisms constituted one of the most frequently investigated domains. The main mechanisms identified were hopelessness, rumination, maladaptive thinking styles, perfectionism, self-criticism, psychological pain, defeat, entrapment, negative future orientation, and fear of failure. These mechanisms were generally associated with higher levels of suicidal ideation, although their relative contribution varied according to the characteristics of the samples, the measures employed, and the variables included in the statistical models.

Hopelessness has been consistently identified as both a direct correlate and an intervening variable between distal contextual stressors (e.g., peer victimization, adverse life events, and academic strain) and suicidal thoughts (4, 17, 18). Intensive longitudinal monitoring has shown that hopelessness fluctuates in real time alongside momentary suicidal desire, rather than behaving as an immutable trait (11). However, multivariate analysis revealed that its direct association is sensitive to adjustment for covariates; although a statistically significant bivariate association was found between hopelessness and scores on the Adult Suicidal Ideation Questionnaire (r = .54), this main effect lost significance after depressive symptoms, perceived burdensomeness, and the corresponding interaction term were included in the model (19).

Both cross-sectional and prospective models demonstrated that repetitive negative thinking (RNT) and rumination correlate with suicidal ideation directly and through multi-step indirect pathways involving childhood adversity, perfectionistic concerns, and perceived strain (20–23). Complementary indirect pathways were also observed in models examining single-item screening proxies (24, detailed in Section 3.5.1). Within these conceptual chains, defeat, entrapment, and depressive affect frequently functioned as sequential mediators (17, 23, 25). However, the independent predictive utility of rumination was not uniform across study designs. In multivariable longitudinal modeling controlling for baseline depressive symptoms, the prospective association of rumination with suicidal ideation was substantially attenuated (23). (Parallel attenuations were observed for momentary passive death desires in complementary assessments; (26, synthesized in Section 3.5.1). These patterns suggest that perseverative thinking operates primarily as a statistical intermediary that covaries closely with underlying depressive distress rather than functioning as an invariant independent predictor of suicidal desire. Methodological variations in outcome operationalization were also evident across the included literature: whereas some investigations evaluated dedicated multi-item ideation inventories (23, 25), others relied on brief screening proxies (24), requiring careful distinction from studies where suicidal rumination itself served as the primary outcome.

Investigations examining perfectionism identified distinct association patterns across its dimensions. Socially prescribed perfectionism and maladaptive concerns were recurrently linked to elevated suicidal ideation through both direct and indirect pathways, whereas self-oriented perfectionism yielded mixed or non-significant relationships (27–29). These indirect associations were frequently accounted for by self-criticism, thwarted belongingness, appearance-based rejection sensitivity, and anti-mattering (21, 23–30). Addressing measurement variations, an assessment utilizing an adapted single-item screener found that socially prescribed perfectionism retained a direct association with ideation, while indirect paths emerged via loneliness rather than anxious rejection sensitivity alone (29). Longitudinal studies added temporal granularity: one three-wave study tracked perfectionism, anti-mattering, acceptance difficulties, and ideation while controlling for baseline distress (30). Another prospective cohort of students facing academic failure observed that although perfectionism preceded subsequent distress, intermediate cognitive variables and ideation were measured concurrently at follow-up, limiting inferences regarding the complete temporal sequence of mediation (23). Finally, educational contexts revealed that parental criticism and evaluative concerns co-occurred with heightened vulnerability, such that elevated personal standards coupled with fear of failure correlated with more frequent suicidal thoughts (31).

Consistent with integrated motivational models, psychological pain, defeat, and entrapment formed a closely linked triad of cognitive–affective processes. Psychological pain was associated with both passive death wishes and active suicidal thoughts; however, the discrepancy between unbearable psychic pain and perceived life meaning was uniquely elevated among students reporting active ideation characterized by explicit intent and lethal consideration (32). In evaluations relying on dedicated multi-item ideation scales, entrapment demonstrated robust positive associations with suicidal ideation, mediating the pathway from perceived defeat and perseverative rumination to acute suicidal thoughts (25, 33). A complementary indirect pathway through entrapment was similarly reported for composite suicide risk using the RIQ-13 (34).

In summary, the aggregated evidence demonstrates that cognitive–affective vulnerability among university students is theoretically conceptualized as perseverative cognitive patterns that covary with heightened negative affect, lower perceived alternatives, and greater self-reported entrapment. While the empirical convergence surrounding hopelessness, maladaptive perfectionism, and entrapment is extensive, current conclusions remain qualified by a high prevalence of concurrent cross-sectional designs, frequent collinearity with major depressive symptoms, and variable psychometric depth between dedicated continuous scales and brief screening proxies.

#### 3.4.2 Emotional mechanisms

The emotional mechanisms identified across the included studies comprised emotion-regulation difficulties, depressive symptoms, anxiety, negative affect, psychological distress, emotional vulnerability, and stress-related responses. In general, greater emotional distress and more pronounced difficulties in regulating negative emotional experiences were associated with higher levels of suicidal ideation.

Emotion-regulation difficulties were examined as both direct correlates and intermediary mechanisms linking stress to suicidal thoughts. Difficulties in identifying, modulating, and accepting emotional distress were consistently associated with elevated suicidal ideation across standardized continuous ideation measures (35). In addition, intensive repeated-measures data indicated that momentary surges in negative affect and emotion-regulation deficits covaried with fluctuations in suicidal ideation over time (35).

Nevertheless, the reviewed evidence does not justify a uniform claim that emotion-regulation difficulties independently predict suicidal ideation across all contexts. A substantial proportion of regulation studies operationalized outcomes not as pure ideation, but as broader suicide-related cognitions, composite risk totals, or inventories combining positive and negative ideation items. Consequently, while deficits in emotion regulation clearly impair distress tolerance, their direct, independent association with specific multi-item ideation scales remains conditionally dependent on co-occurring cognitive-affective factors such as hopelessness and perceived burdensomeness.

Depressive symptom severity demonstrated a robust, ubiquitous concurrent association with suicidal ideation across diverse undergraduate and postgraduate samples evaluated with dedicated continuous ideation inventories (3, 35, 36). In structural equation and multivariable path models, depressive symptoms consistently functioned as a proximal affective correlate, mediating distal vulnerabilities arising from perfectionistic concerns, perseverative rumination, and family dysfunction (20, 23, 37). Among clinical and substance-using student subgroups, baseline depressive symptoms served as a primary vulnerability whose link to suicidal outcomes was channeled through behavioral disinhibition and impulsivity (38).

However, methodological scrutiny reveals critical caveats. First, symptom severity describes a concurrent clinical state rather than a functional explanatory process. Second, potential item-content overlap between standard depression scales (e.g., PHQ-9, BDI-II) and suicide-specific outcomes complicates the interpretation of direct effects unless suicide items are statistically partialed out. Third, design features must be interpreted cautiously: for example, an adjusted association between depressive symptoms and a separately assessed history of suicidal ideation was identified using baseline data (38); despite the overarching prospective design of the parent project, this specific analysis was cross-sectional, precluding causal inference. Longitudinal modeling confirmed that while depressive symptoms predicted subsequent ideation or membership in chronic suicide-risk trajectories (23), depression never fully accounted for the variance in suicidal outcomes. Residual contributions from hopelessness, psychological pain, impulsivity, and thwarted belongingness remained substantial across models.

Negative affect and emotional vulnerability exhibited conditional patterns across samples. Greater negative affect covaried with higher suicidal ideation, whereas positive affect tracked with lower ideation severity in ecological momentary assessments (35). Among university students with disabilities, negative emotion demonstrated positive shared variance with suicidal ideation, while self-acceptance exhibited an inverse link with suicidal desire, with hopelessness acting as a partial mediator or suppressor (39). Emotional vulnerability alone failed to display a significant direct relationship with suicidal ideation in Japanese undergraduates; however, its interaction with depressive symptom severity demonstrated a prominent, recurrent concurrent association with suicidal ideation (36).

Similarly, somatic anxiety, academic test anxiety, and broader psychological strain exhibited less consistent direct associations (20, 40, 41). In multivariable models incorporating multiple emotional and psychiatric indices simultaneously, the independent association of generalized anxiety was frequently attenuated or rendered non-significant (42). Retaining operational specificity is crucial here: an investigation examining negative future disposition, somatic anxiety, general distress, and positive affect as distinct constructs demonstrated that specific affective facets maintain independent associations rather than amalgamating them into a broad depressive composite (41). Their findings confirmed that specific affective facets (such as pessimistic future disposition) maintain independent associations, whereas generalized affective distress frequently operates indirectly through hopelessness or perseverative cognition.

Affective distress and regulatory difficulties rarely accounted for suicidal ideation independently of co-occurring cognitive vulnerabilities. Across multivariable models, generalized emotional distress was frequently attenuated once hopelessness or perseverative thinking was controlled, underscoring the need for repeated-measures designs that account for symptom overlap with depression rating scales.

#### 3.4.3 Behavioral mechanisms

Behavioral mechanisms and related behavioral vulnerabilities included impulsivity, experiential avoidance, nonsuicidal self-injury, previous suicidal behavior, substance-use problems, and reduced engagement in health-promoting behaviors. Compared with cognitive–affective and emotional mechanisms, these variables were examined less frequently as independent correlates and more commonly as mediators, moderators, or indicators of increased vulnerability to suicidal ideation.

The role of impulsivity diverged substantially according to analytical model specifications. Direct associations between trait impulsivity and suicidal outcomes were observed across cross-sectional investigations (12, 43), though effect sizes remained modest when evaluated as an isolated bivariate correlate (44). Importantly, in nursing students, impulsivity was examined in relation to a binary suicidal behavior classification derived from the Beck Scale for Suicide Ideation (BSS) rather than an isolated, continuous ideation frequency metric (43). More prominently, impulsivity operated as a statistically mediated pathway or conditional moderator, exacerbating suicidal vulnerability in the presence of depression, substance-use problems, and obsessive-compulsive symptoms across continuous and structured models (12, 38, 45, 46).

Experiential avoidance was examined as a behavioral response to distressing thoughts and emotions. Greater experiential avoidance was associated with suicidal ideation or moderated associations involving depression and psychopathology across cross-sectional models evaluating ideation-specific measures (47–49). These findings suggest that attempts to suppress or avoid unwanted internal experiences may be relevant to suicidal ideation when accompanied by depression, rumination, childhood adversity, or impaired family functioning. Nevertheless, the cross-sectional nature of these analyses does not establish whether experiential avoidance precedes suicidal ideation or develops in response to persistent psychological distress.

Nonsuicidal self-injury and previous suicidal behavior were recurrent behavioral indicators of elevated vulnerability. University students reporting a history of nonsuicidal self-injury or suicidal behavior generally presented higher levels of suicidal ideation, perceived burdensomeness, loneliness, depression, hopelessness, and experiential avoidance than students without these histories (47–50). These behaviors should be interpreted as markers of vulnerability rather than interchangeable manifestations of suicidal ideation because nonsuicidal self-injury and suicidal thoughts represent conceptually distinct outcomes.

Substance-use problems were also associated with suicidal ideation and broader suicide-related outcomes in several studies. Problematic cannabis use was related to suicidal ideation and suicide-attempt history, particularly in conjunction with impulsivity and childhood trauma (45). Among higher-education students with depression or substance-use problems, impulsivity partly accounted for associations with suicide-related outcomes (38). Other findings indicated that alcohol and other substance-use indicators were associated with suicidal behavior, although depression and hopelessness generally showed more robust associations in adjusted models (43). Because this latter analysis operationalized the outcome as a broad, composite suicidal behavior status (encompassing any reported suicidal desire or previous attempts), substance use in this cohort reflects general vulnerability across the suicidal spectrum rather than a direct predictor of pure suicidal ideation severity alone (43).

Overall, impulsivity and experiential avoidance appeared to be the behavioral mechanisms most closely associated with suicidal ideation. Their contribution was generally embedded within broader pathways involving depression, rumination, psychological distress, substance-use problems, or adverse experiences. Nonsuicidal self-injury and previous suicidal behavior were also important indicators of vulnerability but should not be interpreted as equivalent to suicidal ideation. Because most findings were cross-sectional and based on self-reported behaviors, the temporal direction of these associations remains uncertain.

#### 3.4.4 Interpersonal and contextual mechanisms

Interpersonal and contextual mechanisms included loneliness, perceived burdensomeness, thwarted belongingness, social disconnection, impaired family functioning, adverse childhood experiences, bullying victimization, and stressors originating in academic or digital environments. These factors were generally associated with greater suicidal ideation, either directly or through cognitive and emotional mechanisms such as hopelessness, depression, rumination, and psychological pain.

Perceived burdensomeness and thwarted belongingness were recurrently associated with suicidal ideation across university samples. Under the Interpersonal Theory of Suicide (8), perceived burdensomeness represents the cognitive distortion that one’s existence constitutes an unbearable liability to others, whereas thwarted belongingness denotes an unmet psychological need for social integration and reciprocal connection. In cross-sectional models utilizing psychometrically dedicated ideation instruments, perceived burdensomeness showed robust positive associations with suicidal ideation, remaining significant after adjusting for depressive symptoms, coping competence, and other interpersonal covariates (18, 32). Network analysis further suggested that the central positioning of these interpersonal constructs within symptom clusters may vary according to gender (51).

Adverse family dynamics and childhood maltreatment demonstrated indirect associations with composite suicide risk across multidimensional measures (6, 52–55; detailed in Section 3.5.2), whereas their direct links to separately assessed suicidal ideation were documented primarily in specific subgroups, such as students with disabilities (39).

Academic and technology-related contexts also contributed to the psychological pathways associated with suicidal ideation. Test anxiety, digital stress, academic burnout, and elevated educational expectations were associated with greater suicidal ideation (40, 56, 57). These associations frequently involved emotion-regulation difficulties, psychological pain, hopelessness, rumination, reduced meaning in life, or perfectionism. Therefore, academic and digital stressors appeared to be relevant primarily when co-occurring with, or operating in conjunction with, underlying cognitive–affective mechanisms.

Contextual and interpersonal stressors—including social disconnection, burdensomeness, and family strain—demonstrated robust bivariate links to suicidal ideation, yet their adjusted contributions operated largely through intermediate affective states such as hopelessness, psychache, and depressive affect. Consequently, environmental stressors appear to heighten vulnerability by activating negative cognitive-affective appraisals rather than through isolated direct pathways. Their associations were frequently indirect or conditional, operating through hopelessness, depression, rumination, psychological pain, experiential avoidance, and loneliness. The findings support the relevance of contextual conditions while indicating that their relationship with suicidal ideation is closely intertwined with individual psychological mechanisms.

#### 3.4.5 Protective mechanisms

Protective mechanisms comprised self-compassion, mindfulness, resilience, coping competence, meaning and purpose in life, reasons for living, gratitude, self-acceptance, positive affect, and perceived social support. Across evaluations relying on direct, multi-item ideation inventories, these internal and relational resources were generally associated with lower suicidal ideation or attenuated the positive associations between cognitive-affective risk factors and suicidal thoughts. Given the predominance of cross-sectional designs, these results represent statistical protective associations rather than demonstrated preventive effects.

Self-compassion and mindfulness were examined as internal cognitive-affective resources against hopelessness, negative life events, and perfectionistic concerns. In evaluations using standardized continuous symptom scales, self-compassion showed inverse bivariate associations with suicidal thoughts (28); however, although a buffering role against maladaptive perfectionism was hypothesized, formal statistical moderation was not established in the reported models (28). Similarly, mindfulness attenuated the positive association between entrapment and suicidal ideation and was inversely correlated with perseverative rumination and psychological distress (25). Notably, empirical comparisons indicated that mindfulness did not function as an omnibus protective factor; rather, its moderating buffer against hopelessness was driven specifically by its compassion facet (58).

Positive affect was similarly associated with lower suicidal ideation, whereas expressive suppression and negative affect correlated with heightened vulnerability in intensive repeated-measures data (35). In contrast, although cognitive reappraisal was proposed as an adaptive buffer in one cross-sectional report (56), statistical reporting inconsistencies preclude its classification as a confirmed protective correlate. Intensive repeated-measures data confirmed that momentary purpose in life and positive affect covaried inversely with real-time fluctuations in suicidal ideation (35). These observations suggest that active emotional acceptance and cognitive re-evaluation are more adaptive than avoidant or suppressive coping responses.

Resilience and coping competence demonstrated consistent inverse associations with psychological vulnerability. Greater resilience attenuated associations involving perseverative rumination and borderline personality features (59), while coping competence statistically moderated (attenuated) the cross-sectional association between perceived burdensomeness and suicidal ideation (18). In academic and achievement settings, psychological capital similarly buffered the relationship between test anxiety and suicidal thoughts (40). However, because resilience was typically examined within multivariable models alongside broader distress indices, isolating its independent contribution remains challenging.

Meaning in life, purpose, and reasons for living were recurrently associated with lower suicidal ideation across dedicated multi-item instruments. Presence of life meaning attenuated the adverse impact of failure mindset, psychological pain, and internal entrapment across validated multi-item scales (32, 33, 60). Furthermore, reasons for living accounted for substantial variance in mitigating active suicidal desire (33).

Gratitude, self-acceptance, and perceived social support represented complementary internal and relational resources. Gratitude attenuated the cross-sectional relationship between hopelessness and suicidal ideation (17), while self-acceptance was negatively associated with suicidal thoughts among university students with disabilities (39). Support from family, peers, and significant others consistently buffered associations involving impostor feelings and depressive symptoms across multi-item ideation models (37). Finally, health-promoting behaviors, such as physical activity, showed indirect inverse associations with suicidal ideation through lower rumination and depressive mood (61).

In summary, protective resources within the primary evidence base operate at multiple levels of the psychological pathway: inverse or conditional associations involving psychological resources were reported in selected analysis, but findings varied by construct, outcome measure, and statistical adjustment. Internal resources such as self-compassion, mindfulness, and presence of meaning primarily exhibited statistical buffering associations against situational strain rather than uniform protective effects. Meaning, gratitude, and reasons for living foster adaptive future orientation, whereas perceived social support attenuates interpersonal disconnection. Rather than eliminating risk entirely, these mechanisms primarily attenuate the statistical magnitude of vulnerability cascades associated with suicidal ideation.

### 3.5 Synthesis of complementary and distinct outcome metrics

Complementary findings were organized according to the outcome analyzed in the primary study: broad death/self-harm screening indicators, multidimensional composite suicide-risk scores, proximal suicide-related cognitive constructs, and relevant behavioral-history profiles within otherwise eligible studies. Their inclusion contextualizes the principal ideation-focused question without treating these outcomes as equivalent to separately assessed suicidal ideation. These categories describe the synthesis and are not presented as prospectively specified outcome hierarchies.

#### 3.5.1 Broad indicators of death and self-harm thoughts

Several investigations relied on single-item screening proxies or broad indicators of self-harm, which do not assess suicidal frequency, severity, or explicit intent. One cross-sectional investigation examined the single death/self-harm item of the PHQ-9 within a clinical help-seeking student sample, reporting indirect associations involving social anxiety, diminished self-esteem, and hopelessness (62). Extending this observation, a prospective cohort study administered the PHQ-9 suicide proxy (item 9) at longitudinal follow-up, which captures broad thoughts of death or self-harm without differentiating explicit suicidal intent. In the reported path model, perceived burdensomeness demonstrated both direct and indirect prospective associations with follow-up death/self-harm thoughts through hopelessness (63). In contrast, the independent direct path from thwarted belongingness to the follow-up outcome was nonsignificant. Thwarted belongingness operated exclusively through an indirect association via hopelessness, alongside a statistically significant perceived-burdensomeness × thwarted-belongingness interaction (PB × TB) on follow-up scores (63). Importantly, this interaction term reflects a conditional multiplicative effect that must be distinguished from the main effect of thwarted belongingness. Similarly, in models examining single-item screening proxies, childhood sexual abuse was associated with death/self-harm thoughts via rumination, with perceived social support statistically buffering this indirect pathway on the SCL-90 proxy measure (24).

Using distinct screening items, an analytical survey evaluated a single DSM-5 self-harm cognition item, identifying associations with fear of negative evaluation, negative urgency, and low perseverance (64), while a cross-national investigation evaluated self-harm and suicidal thoughts using a single-item DSM-5 screening indicator across four countries within a multivariable model incorporating emotional stability, repetitive negative thinking, and depressive symptoms (65). Other studies operationalised passive or indirect death-related thoughts: one report evaluated an isolated ‘death-as-release’ item (66), whereas an intensive ecological momentary assessment tracked separate momentary ratings of ‘wishes to die’ versus ‘wishes to live’ (26). In the latter, the prospective link between perseverative rumination and passive death wishes was substantially attenuated once concurrent depressive symptoms were modelled. These findings emphasise that passive death wishes and inverted ‘wish-to-live’ items reflect distinct motivational stages that cannot be treated as psychometrically interchangeable with active suicidal ideation.

#### 3.5.2 Multidimensional composite suicide-risk scores

Studies employing composite instruments—such as the Suicidal Behaviors Questionnaire-Revised (SBQ-R) or the Inventory of Suicide Orientation (ISO-30)—were analysed strictly as associations with multidimensional risk composites rather than pure ideation. For example, in prospective multi-wave modeling among sexual and gender minority students, baseline negative core beliefs directly predicted subsequent composite SBQ-R scores; however, hypothesized indirect pathways through avoidance and perseverative thinking were nonsignificant, and self-compassion was removed during measurement trimming due to overlap with baseline beliefs (67). An investigation examining loneliness and impostor phenomena among doctoral researchers using composite SBQ-R totals noted that supervisor communion was rendered non-significant in final adjusted models (68). In a trajectory analysis, developmental trajectories of composite suicide risk were modelled over time, linking persistent high-risk trajectories to baseline hopelessness, depressive symptoms, adverse life events, and diminished reasons for living (69).

Additional composite analysis evaluated family functioning (52), obsessive-compulsive symptom dimensions alongside motor impulsivity (46), interpersonal needs alongside depressive affect in developing higher-education contexts (70), and pathways connecting defeat, repetitive negative thinking, and entrapment (34). Methodological caution is particularly warranted for findings derived from the ISO-30: because risk domain subscales (such as hopelessness or coping deficits) constitute component items of the composite total, bivariate correlations partly reflect shared item content and circular method variance (12, 49). Consequently, findings involving composite scales cannot be combined with dedicated ideation subscales to claim independent empirical replication. Similarly, documented associations involving religious affiliation (71) pertained specifically to composite risk totals and should not be inferred as protective effects against active suicidal ideation.

#### 3.5.3 Related cognitive outcomes and behavioral-history profiles

A subset of eligible investigations evaluated proximal cognitive constructs or retrospective behavioral histories. Specifically, suicide cognitions were evaluated using the 16-item Suicide Cognitions Scale–Revised (SCS-R), demonstrating a serial indirect pathway from maladaptive thinking through emotion dysregulation and loneliness to proximal suicide beliefs, rather than predicting direct suicidal ideation frequency (72). In parallel, suicidal rumination was operationalised specifically as the terminal outcome in a separate investigation (73). These investigations capture perseverative suicide-related thinking rather than verifying whether general dysregulation predicts a separately assessed ideation outcome. Similarly, studies using the Positive and Negative Suicide Ideation (PANSI) inventory required explicit separation of protective buffers (positive ideation) from active distress (negative ideation) before attributing associations to suicidal risk (56, 74, 75). When positive items are reverse-scored into global composites or analyzed at the item-network level without isolating pure suicidal desire, findings capture composite suicide-related vulnerability rather than dedicated ideation severity (56, 74).

In a laboratory-based observational study administering both the SBQ-R and an implicit death/suicide response-latency task, self-compassion was inversely associated with self-reported composite suicide risk, but was non-significantly associated with the implicit suicide task (76). These findings concern distinct measurement modalities and do not establish a differential functional mechanism operating selectively on conscious awareness. Finally, latent class analysis delineated historical profiles of nonsuicidal self-injury, ideation, and suicide attempts (53, 54), while separate cohort comparisons contrasted students selected exclusively by suicide attempt status (77).

## 4 Discussion

The retrieved empirical studies examined candidate psychological variables across cognitive–affective, emotional, behavioral, interpersonal, and protective domains. Associations varied considerably according to outcome definitions, sample characteristics, and multivariable covariate adjustments. Rather than confirming a comprehensive explanatory model or demonstrating superiority over conventional psychiatric categories, this synthesis characterizes candidate processes that warrant further prospective evaluation while identifying substantial methodological boundaries within the university literature. A mechanism-focused perspective provides a valuable heuristic for conceptualizing how psychological vulnerabilities operate in higher education. Individual variables rarely functioned in isolation; across analytical models, cognitive vulnerabilities, affective distress, and interpersonal strain frequently interacted within complex indirect pathways. However, the predominance of cross-sectional designs, convenience sampling, and reliance on self-report inventories prevent determining whether these processes temporally precede suicidal thoughts or represent concurrent markers of general student distress.

### 4.1 Interpretation of cognitive–affective mechanisms

Hopelessness emerged as one of the most consistently investigated mechanisms associated with suicidal ideation. Its relevance across culturally diverse university populations suggests that negative expectations regarding the future may represent a shared cognitive vulnerability extending beyond specific diagnostic categories (17, 18, 62). Importantly, hopelessness did not operate exclusively as a direct correlate. It frequently appeared as an intermediary mechanism connecting bullying victimization, social anxiety, negative life events, depression, perceived burdensomeness, and rumination with suicidal ideation (4, 5, 62).

This finding is broadly consistent with the Three-Step Theory, which proposes that psychological pain and hopelessness contribute to the emergence of suicidal ideation, particularly when pain is accompanied by diminished expectations for improvement (9). However, the reviewed evidence suggests that hopelessness among university students may be shaped by multiple academic, interpersonal, and emotional experiences. Consequently, hopelessness should not be interpreted solely as a symptom of depression but as a potentially transdiagnostic cognitive mechanism associated with how students evaluate current distress and future alternatives.

Rumination and repetitive negative thinking may contribute to the persistence of this cognitive vulnerability. The included studies showed that rumination was associated with suicidal ideation through pathways involving depression, hopelessness, entrapment, perfectionism, and adverse experiences (20, 22–25). Ecological momentary assessment findings further indicated that short-term increases in rumination were associated with passive suicidal ideation (26). These findings are compatible with a transdiagnostic interpretation of repetitive negative thinking as a process that may prolong emotional distress across different psychological conditions.

Nevertheless, rumination was not independently associated with suicidal ideation in every adjusted model. This variability suggests that its relevance may depend on the content of repetitive thinking, the presence of depression or hopelessness, and the student’s perceived capacity to resolve or escape distress. From this perspective, repetitive thinking appears more consequential when statistical models account for internal entrapment and depressive affect rather than reflecting thought frequency alone. In academic contexts, perfectionistic concerns and test-related anxiety covary with suicidal thoughts (40), though cross-sectional designs prevent inferring that academic pressures directly precipitate suicidal ideation.

Perfectionism findings further illustrate the need to distinguish dimensions and outcomes. In an evaluation using an adapted single ideation item, socially prescribed perfectionism had a direct association, whereas self-oriented perfectionism did not; loneliness appeared in the reported indirect associations, but the rejection-sensitivity-only indirect association was not significant (29). This supports a dimension-specific interpretation, without establishing that externally imposed standards cause ideation or that self-oriented standards are harmless in every context. Evidence from self-criticism models provides related observations, but limited confounder adjustment restricts conclusions about unique contributions (27).

The longitudinal findings (23, 30) strengthen this interpretation by suggesting that perfectionistic concerns may precede subsequent suicidal ideation through interpersonal disconnection, difficulty accepting past experiences, rumination, or depression. Even so, these findings should not be generalized to all forms of perfectionism. Striving for high standards may not necessarily represent a risk-related mechanism unless accompanied by self-criticism, perceived failure, inflexibility, or social disconnection.

Observed associations involving entrapment and defeat align conceptually with ideation-to-action models—most notably the Integrated Motivational–Volitional model (10) and the Three-Step Theory (9)—which position perceptions of unbearable distress without perceived rescue at the core of suicidal desire. Within this framework, defeat and repetitive thinking appear to foster acute distress primarily when channelled through feelings of internal or external entrapment. Rather than validating these theoretical architectures in their entirety, the present observational evidence demonstrates that cognitive perceptions of entrapment serve as critical cognitive-affective markers that consistently covary with active suicidal ideation across student samples.

### 4.2 Interpretation of emotional mechanisms

Depressive symptoms were frequently included across the reviewed multivariable models, consistently emerging as prominent concurrent correlates of suicidal outcomes. However, the statistical interpretation of depressive severity requires particular caution when depression rating scales (e.g., PHQ-9, BDI-II) contain suicide- or self-harm-related items. In the absence of item-level partialing, reported associations partly reflect shared measurement content rather than distinct functional mechanisms. Statistical adjustment across concurrent psychiatric indices also shows that generalized negative affect frequently attenuates once specific cognitive appraisals are modeled.

A positive statistical interaction was observed between depressive symptoms and emotional vulnerability despite a non-significant main association of vulnerability (36). In parallel, in multivariable models that incorporated somatic symptoms, general distress, and positive affect as discrete constructs, generalized affective distress operated indirectly rather than as an independent mediator of depressive symptoms (41).

Emotion-regulation findings require additional outcome distinctions. While one investigation evaluated suicide-specific cognitions rather than direct ideation (72), other reports relied on mixed positive and negative ideation totals or broader composite risk indices (56, 74). These results may inform hypotheses about suicide-related distress, but they do not establish consistent associations between specific regulation strategies and separately measured suicidal thoughts. Accordingly, the empirical evidence does not warrant a general conclusion that cognitive reappraisal or positive affect uniformly buffered suicidal ideation across the included studies.

### 4.3 Interpretation of behavioral mechanisms

Behavioral mechanisms appeared to moderate or contextualize the concurrent associations between cognitive–emotional distress and suicidal ideation. Impulsivity was the most frequently examined behavioral mechanism, although its role varied across studies. In some models, impulsivity was directly associated with suicidal ideation, whereas in others it mediated or intensified associations involving depression, obsessive-compulsive symptoms, substance-use problems, cannabis use, and fear of negative evaluation (38, 45, 46, 64). This variability suggests that impulsivity may be particularly relevant when combined with emotional distress, impaired inhibition, or other psychological vulnerabilities rather than functioning as an isolated correlate.

Experiential avoidance represented another potentially important behavioral mechanism. Greater avoidance of unwanted internal experiences was associated with suicidal ideation or moderated associations involving depression, family functioning, psychopathology, childhood adversity, and suicide-related outcomes (47, 48). From a transdiagnostic perspective, experiential avoidance may restrict students’ ability to process distressing emotions and respond adaptively to adverse experiences. Nevertheless, the predominance of cross-sectional designs prevents determining whether avoidance precedes suicidal ideation or emerges as a response to sustained psychological distress.

Nonsuicidal self-injury and previous suicidal behavior were strongly correlated with suicidal outcomes (47–50). From an ideation-to-action theoretical framework (8, 9), these behavioral factors are conceptualized not as generative mechanisms of suicidal desire, but as potential indicators of acquired capability for suicide, hypothesizing that painful and provocative experiences habituate individuals to physical pain and diminish the fear of death (8, 9). However, this acquired-capability proposition represents an external theoretical hypothesis that was not directly tested or confirmed by the observational evidence synthesized in this review. In prospective trajectory analysis, reported associations pertained to membership in persistent high composite suicide-risk trajectories rather than transitions from isolated ideation to lethal action (69).

Substance-use problems were associated with suicidal ideation or broader suicide-related outcomes, particularly when accompanied by impulsivity, depression, or childhood trauma (38, 43, 45). Nevertheless, depression and hopelessness generally showed more robust associations than substance-use indicators in adjusted models. Therefore, substance use may be better understood as a contextualized behavioral vulnerability that interacts with emotional and cognitive mechanisms rather than as a sufficient explanation for suicidal ideation.

### 4.4 Interpretation of interpersonal and contextual mechanisms

Perceived burdensomeness, loneliness, and related experiences can be considered in relation to interpersonal accounts of suicide (8). However, loneliness, thwarted belongingness, feelings of not mattering, and perceived burdensomeness are distinct constructs rather than interchangeable indicators. Ideation-specific evidence supported conditional associations involving burdensomeness, hopelessness, and coping competence (18), while loneliness accounted for indirect vulnerability within multidimensional perfectionism models (29).

Longitudinal findings illustrate the importance of distinguishing bivariate correlations, main path effects, and multiplicative interaction terms (63). Although both constructs were theoretically conceptualized as prospective predictors, the final structural path model revealed that the direct path from thwarted belongingness to follow-up death/self-harm thoughts was statistically nonsignificant (β = .149), operating instead via hopelessness and through a significant PB × TB interaction (β = .381) (63). Consequently, the nonsignificant independent direct path from thwarted belongingness cannot be interpreted as a standalone direct effect solely on the basis of the significant interaction term. The reported indirect association through hopelessness also warrants caution because hopelessness and the follow-up outcome were measured concurrently at T2 (63). Methodologically, the PHQ-9 item 9 outcome does not differentiate thoughts of death from self-harm thoughts or establish suicidal intent. Accordingly, these findings provide complementary evidence concerning a broad death/self-harm indicator rather than confirmation of an ideation-specific causal pathway (63).

Contextual findings, including bullying and childhood sexual abuse, suggest useful questions about the conditions accompanying distress and ideation (4, 24). Contextual exposure, a putative psychological process, and an outcome nevertheless occupy different conceptual roles. Their inclusion in one statistical model does not show that the proposed process explains the exposure’s effect. Family-functioning findings based on composite risk scores likewise broaden the contextual description without proving ideation-specific pathways (49, 52). Associations between subscales and totals from the same instrument require particular caution because shared components can produce structural overlap.

The geographical diversity of the reports does not demonstrate cultural moderation. Differences between countries may reflect recruitment, instruments, assessment windows, sample composition, or analytical adjustment. Explanations invoking collectivism, family honor, individualism, or autonomy should therefore be treated as hypotheses for direct comparative research, not as findings of this synthesis. Similarly, claims about gender-specific pathways or undergraduate versus postgraduate trajectories require formal subgroup analysis or interaction tests and cannot be inferred from the demographic composition of separate studies.

### 4.5 Protective mechanisms and integrative interpretation

Protective mechanisms appeared to display statistically attenuating or moderating patterns across different levels of psychological vulnerability, rather than simply representing the absence of risk-related mechanisms. Self-compassion, mindfulness, resilience, coping competence, meaning and purpose in life, reasons for living, gratitude, self-acceptance, self-esteem, positive affect, and perceived social support were generally associated with lower suicidal ideation or weaker associations between risk-related mechanisms and suicidal ideation.

Mindfulness and adaptive emotion regulation showed conditionally inverse associations with suicidal distress. Mindfulness attenuated the statistical association between entrapment and suicidal ideation and was negatively correlated with rumination (25, 58). However, internal self-regulatory resources cannot be conceptualized as uniform, invariant protective factors across all contexts. Specifically, empirical findings demonstrated that global mindfulness did not function as an omnibus buffer; rather, its moderating association against hopelessness was driven specifically by its compassion facet (58). In parallel, in longitudinal structural modeling among sexual and gender minority undergraduates, self-compassion was excluded from the final structural model during measurement specification due to item overlap with negative core beliefs (67); its omission reflects psychometric refinement rather than an estimated null protective effect on suicidality (67).

Resilience and coping competence were also associated with lower vulnerability. Greater resilience attenuated associations involving rumination, depression, borderline personality features, and psychological strain, while coping competence weakened the association between perceived burdensomeness and suicidal ideation (18, 57, 71). However, resilience should not be interpreted as a fixed individual trait that places responsibility solely on students. Its expression may depend on the availability of supportive relationships, institutional resources, and opportunities to develop adaptive coping strategies.

Greater meaning was inversely associated with suicidal ideation and attenuated cross-sectional associations involving failure mindset, psychological pain, and entrapment (33, 60). Although adaptive buffering against digital stress was hypothesized in a mixed-outcome evaluation (56), unresolved reporting contradictions in the original statistical presentation preclude drawing directional inferences regarding cognitive reappraisal in that cohort. Ecological momentary assessment findings further indicated that purpose in life was associated with lower suicidal ideation over time (35). Longitudinal evidence suggested that purpose in life partly accounted for the association between depressive symptoms and subsequent death/self-harm thoughts assessed via screening indicators at follow-up (77). Reasons for living similarly differentiated lower-risk courses from persistent high composite suicide-risk trajectories (69).

Gratitude attenuated the association between hopelessness and suicidal ideation (17), while self-esteem was inversely associated with broad death/self-harm thoughts through lower hopelessness in screening data (64). Meaning in life mediated prospective associations involving follow-up screening proxies (78), and resilient coping buffered psychological strain in models examining suicidal rumination (73).

Perceived social support extended these protective processes beyond the individual level. Support from family, peers, and significant others attenuated associations involving childhood adversity, impostor feelings, and psychological strain across cross-sectional models (24, 37, 43, 73).

Taken together, the five domains identified in this review can be understood as an interconnected transdiagnostic framework. Interpersonal and contextual stressors may coincide with cognitive–affective vulnerabilities such as hopelessness, rumination, perfectionistic concerns, psychological pain, defeat, and entrapment. Emotional mechanisms, including depression, negative affect, anxiety, and emotion-regulation difficulties, may intensify these associations, while impulsivity and experiential avoidance may shape behavioral responses to distress. Protective mechanisms may operate across these domains by attenuating negative self-appraisals, supporting emotional regulation, strengthening social connection, and preserving future-oriented meaning.

This framework is consistent with a transdiagnostic perspective in which shared psychological processes extend across diagnostic categories and contribute to multiple forms of distress (7). It also complements suicide-specific theories emphasizing psychological pain, hopelessness, burdensomeness, social disconnection, and the perceived absence of alternatives (8, 9). However, the framework proposed here should be interpreted as an evidence-informed conceptual synthesis rather than as an empirically validated causal or temporal model.

### 4.6 Theoretical and practical implications

This review supports a transdiagnostic research agenda requiring explicit operational distinctions among active suicidal ideation, passive death wishes, proximal suicide cognitions, and behavioral outcomes. While compatible with contemporary theories, these observational findings do not validate a comprehensive causal model or the transition from ideation to lethal action. Definitive prospective validation requires temporally ordered multi-wave designs, baseline outcome adjustment, and rigorous psychiatric confounder control.

For university services, the findings identify topics that may be relevant to a broader psychological assessment, including hopelessness, repetitive negative thinking, perceived burdensomeness, loneliness, and available coping resources. These observations do not validate a screening algorithm or establish that any combination improves identification beyond direct assessment of suicidal thoughts and behavior. Clinical decisions should not be based on network centrality, a composite score interpreted as pure ideation, or the presence of a single candidate mechanism.

Although observational evidence from this review cannot establish intervention efficacy, the identified candidate mechanisms conceptually correspond to distinct targets addressed across different tiers of intervention evaluated in higher education and young adult populations. At the psychotherapeutic level, difficulties in emotion regulation and perseverative negative thinking represent core targets of Dialectical Behavior Therapy skills training (DBT-ST) (79) and Acceptance and Commitment Therapy (ACT) (80), which focus on distress tolerance and psychological flexibility rather than categorical symptom reduction. Distinct from general psychotherapies, suicide-specific clinical protocols such as the Collaborative Assessment and Management of Suicidality (CAMS) (81) and the Safety Planning Intervention (SPI) (82) specifically target suicidal cognitions, drivers of despair, and acute crisis stabilization. At the campus and community level, gatekeeper training programs, such as Question, Persuade, Refer (QPR) (83), aim to mitigate social isolation and facilitate timely service utilization by training peers and institutional staff. Importantly, because intervention studies in university students demonstrate heterogeneous effect sizes and context-dependent outcomes, evidence-informed campus policies should combine standardized direct screening with supportive psychoeducational environments, avoiding assuming uniform preventive effects or placing the responsibility for psychological resilience solely on students.

### 4.7 Strengths, limitations, and future research

This review synthesized 68 recent studies retrieved from four major information sources. Strengths of this systematic review include adherence to PRISMA 2020 guidelines, independent dual screening with consensus resolution, design-specific JBI critical appraisal, and explicit stratification between cross-sectional and repeated-measures designs. Organizing findings across five transdiagnostic domains provides an integrative framework extending beyond categorical diagnoses.

Several limitations in the primary literature constrain these conclusions. Most included studies employed analytical cross-sectional designs (85.3%), convenience sampling, and self-report measures, precluding causal inference, temporal ordering, and adequate control for common-method variance. Potential sample overlap across studies from identical cohorts and inconsistent control for psychiatric confounders (notably depression severity) further qualify the findings. Crucially, measurement heterogeneity across suicidal outcomes is substantive: multi-item ideation scales, single-item screening proxies, broad self-harm thoughts, proximal cognitions, and composite risk inventories capture distinct constructs with varying recall windows. In composite measures (e.g., SBQ-R, ISO-30), ideation is conflated with past behavior, meaning apparent consistency cannot be treated as replication. These challenges align with recent meta-analytic evidence documenting substantial operational heterogeneity across higher education cohorts.

At the review level, the predefined search strategy, restriction to peer-reviewed literature in four databases, and lack of prospective protocol registration leave publication and availability biases possible. Future research should prespecify outcome constructs to distinguish passive death wishes, active ideation, and behavioral enactment. Prospective and intensive repeated-measures studies must temporally separate exposures, mediators, and outcomes while accounting for baseline levels and missing data. Finally, intervention research should test whether deliberately modifying candidate transdiagnostic mechanisms directly reduces suicidal ideation.

## 5 Conclusion

This systematic review synthesized empirical evidence on transdiagnostic psychological variables associated with suicidal ideation among university students across cognitive–affective, emotional, behavioral, interpersonal, and protective domains. Primary ideation-specific findings highlighted recurrent associations with hopelessness, repetitive negative thinking, perfectionistic concerns, depressive symptoms, and perceived burdensomeness, with associations frequently attenuated or rendered conditional after covariate adjustment. A complementary contribution was the independent audit of single-item screening proxies, proximal suicide cognitions, and composite suicide-risk scores, which contextualize general suicide-related distress but are not psychometrically equivalent to dedicated suicidal ideation scales.

The predominance of cross-sectional observational designs and substantive outcome heterogeneity preclude causal inferences, temporal sequencing, or conclusions regarding transitions to suicidal behavior. Accordingly, the proposed multi-domain framework organizes candidate psychological processes for prospective hypothesis testing rather than validating a comprehensive causal model. Future investigations should employ preregistered longitudinal and intensive repeated-measures designs with standardized, ideation-specific outcomes to determine whether modifying these cognitive and interpersonal processes yields meaningful reductions in suicidal thoughts.

## Data Availability

The original contributions presented in the study are included in the article/**Supplementary Material**. Further inquiries can be directed to the corresponding author/s.
