# Supplementary material for: Beyond diagnostic categories: transdiagnostic psychological mechanisms associated with suicidal ideation among university students: a systematic review (2021–2025)
Source: Front Psychiatry. 2026 Oct 21;17:1972940. doi: 10.3389/fpsyt.2026.1972940 (PMC13731204; doi:10.3389/fpsyt.2026.1972940)
Supplement: Supplementary file 1 [file Table2.docx]

| **Section and Topic** | **Item #** | **Checklist item** | **Location where item is reported** |
| --- | --- | --- | --- |
| **TITLE** | | |  |
| Title | 1 | Identify the report as a systematic review. | Title (page 1; indicated as "A Systematic Review (2021–2025)") |
| **ABSTRACT** | | |  |
| Abstract | 2 | See the PRISMA 2020 for Abstracts checklist. | Structured Abstract: Background, Methods, Results, Discussion |
| **INTRODUCTION** | | |  |
| Rationale | 3 | Describe the rationale for the review in the context of existing knowledge. | Section 1, Introduction: public-health rationale, theoretical context, fragmentation of the literature and measurement heterogeneity. |
| Objectives | 4 | Provide an explicit statement of the objective(s) or question(s) the review addresses. | Final paragraph of Section 1; Section 2.2, Review question and eligibility framework: review objective and PECO-based question. |
| **METHODS** | | |  |
| Eligibility criteria | 5 | Specify the inclusion and exclusion criteria for the review and how studies were grouped for the syntheses. | Sections 2.2 and 2.4: population, psychological exposures, eligible outcomes, publication period, designs and exclusions. Sections 2.6 and 2.8: outcome-based grouping and five analytical domains. Section 2.2 explicitly states that the primary/complementary hierarchy was not prospectively specified. |
| Information sources | 6 | Specify all databases, registers, websites, organisations, reference lists and other sources searched or consulted to identify studies. Specify the date when each source was last searched or consulted. | Section 2.3: Scopus, Web of Science Core Collection, PubMed and PsycINFO via EBSCOhost; final search date reported as December 18, 2025. The manuscript states that no supplementary reference-list, citation-index or grey-literature searches were performed. |
| Search strategy | 7 | Present the full search strategies for all databases, registers and websites, including any filters and limits used. | Section 2.3 and Table 1: database-specific search strings; MeSH and title/abstract terms in PubMed; free-text TX in PsycINFO, TITLE-ABS-KEY in Scopus and TS in Web of Science. Section 2.3 reports the publication window, journal-article restriction and absence of language restrictions. Full interface-specific filter settings are not separately documented. |
| Selection process | 8 | Specify the methods used to decide whether a study met the inclusion criteria of the review, including how many reviewers screened each record and each report retrieved, whether they worked independently, and if applicable, details of automation tools used in the process. | Section 2.5 (Independent dual screening of titles, abstracts, and full texts; third-reviewer consensus resolution) |
| Data collection process | 9 | Specify the methods used to collect data from reports, including how many reviewers collected data from each report, whether they worked independently, any processes for obtaining or confirming data from study investigators, and if applicable, details of automation tools used in the process. | Section 2.6 (Standardized piloted Excel matrix; extraction by one reviewer and independent verification by a second reviewer; third-reviewer consensus) |
| Data items | 10a | List and define all outcomes for which data were sought. Specify whether all results that were compatible with each outcome domain in each study were sought (e.g. for all measures, time points, analyses), and if not, the methods used to decide which results to collect. | Section 2.2, Section 2.6, and Table 2 (Separately assessed suicidal ideation vs. complementary outcomes; measurement instruments, recall periods, and specific extracted metrics) |
|  | 10b | List and define all other variables for which data were sought (e.g. participant and intervention characteristics, funding sources). Describe any assumptions made about any missing or unclear information. | Section 2.6 and Table 2 (Study characteristics, country, sample size, designs, candidate mechanisms, and statistical parameters) |
| Study risk of bias assessment | 11 | Specify the methods used to assess risk of bias in the included studies, including details of the tool(s) used, how many reviewers assessed each study and whether they worked independently, and if applicable, details of automation tools used in the process. | Section 2.7 (Methodological quality assessment; design-specific JBI critical appraisal checklists for cross-sectional and cohort/EMA studies; independent dual appraisal) |
| Effect measures | 12 | Specify for each outcome the effect measure(s) (e.g. risk ratio, mean difference) used in the synthesis or presentation of results. | Section 2.6, Section 2.8, Table 2, and Table 3 (Correlation coefficients $r$, standardized path coefficients $\beta$, odds ratios $OR$, and statistical significance thresholds) |
| Synthesis methods | 13a | Describe the processes used to decide which studies were eligible for each synthesis (e.g. tabulating the study intervention characteristics and comparing against the planned groups for each synthesis (item #5)). | Section 2.6, Section 2.8, and Table 2 note (Classification into primary synthesis [$k = 39$], complementary synthesis [$k = 34$], and overlapping contributions) |
|  | 13b | Describe any methods required to prepare the data for presentation or synthesis, such as handling of missing summary statistics, or data conversions. | Section 2.6, Section 2.7, and Section 2.8 (Tabulation, metric standardization, and JBI percentage score conversion) |
|  | 13c | Describe any methods used to tabulate or visually display results of individual studies and syntheses. | Section 2.8; Table 2 (Study characteristics); Table 3 (Mechanism synthesis); Figure 1 (PRISMA flow diagram); Figure 2 (Transdiagnostic conceptual framework) |
|  | 13d | Describe any methods used to synthesize results and provide a rationale for the choice(s). If meta-analysis was performed, describe the model(s), method(s) to identify the presence and extent of statistical heterogeneity, and software package(s) used. | Section 2.1 and Section 2.8 (Rationale for narrative synthesis over meta-analysis due to substantive conceptual, operational, and statistical heterogeneity) |
|  | 13e | Describe any methods used to explore possible causes of heterogeneity among study results (e.g. subgroup analysis, meta-regression). | Section 2.8, Section 3.4, and Section 3.5 (Qualitative subgroup contrasts across measurement formats, study designs [cross-sectional vs. longitudinal], and multivariable adjustments) |
|  | 13f | Describe any sensitivity analyses conducted to assess robustness of the synthesized results. | Section 2.7, Section 2.8, and Section 3.3 (Narrative evaluation of robustness informed by methodological quality tiers: high [≥80%], moderate [50–79%], and low [<50%]) |
| Reporting bias assessment | 14 | Describe any methods used to assess risk of bias due to missing results in a synthesis (arising from reporting biases). | Section 2.8 (Data synthesis: retention of positive, negative, mixed, and non-significant findings to minimize selective reporting bias); Section 4.7 (Limitations) |
| Certainty assessment | 15 | Describe any methods used to assess certainty (or confidence) in the body of evidence for an outcome. | Section 2.7 (Methodological quality thresholds: high [≥80%], moderate [50–79%], low [<50%]); Section 3.3; Supplementary Table S1 |
| **RESULTS** | | |  |
| Study selection | 16a | Describe the results of the search and selection process, from the number of records identified in the search to the number of studies included in the review, ideally using a flow diagram. | Section 3.1 and Figure 1 (327 records identified, 143 duplicates removed, 184 screened, 96 excluded, 88 full-text reports assessed, 20 excluded, and 68 studies included) |
|  | 16b | Cite studies that might appear to meet the inclusion criteria, but which were excluded, and explain why they were excluded. | Section 2.5, Section 3.1, and Figure 1 (Explicit methodological reasons and nominal citations for full-text exclusions: Frey et al., Alwan et al., Antúnez et al., n = 20) |
| Study characteristics | 17 | Cite each included study and present its characteristics. | Section 3.2 and Table 2 (Detailed characteristics of all 68 included empirical studies, including country, sample size, design, mechanisms, instruments, and main findings) |
| Risk of bias in studies | 18 | Present assessments of risk of bias for each included study. | Section 3.3 and Supplementary Table S1 (Item-level JBI ratings and descriptive quality stratification: 50 high [73.5%], 17 moderate [25.0%], 1 low [1.5%) |
| Results of individual studies | 19 | For all outcomes, present, for each study: (a) summary statistics for each group (where appropriate) and (b) an effect estimate and its precision (e.g. confidence/credible interval), ideally using structured tables or plots. | Table 2, Table 3, and Sections 3.4–3.5 (Study-level effect directions, correlational coefficients, standardized path parameters, and narrative summaries) |
| Results of syntheses | 20a | For each synthesis, briefly summarise the characteristics and risk of bias among contributing studies. | Section 3.2, Section 3.3, Table 2, and Table 3 (Summaries of contributing sample designs, measurement characteristics, and quality distribution across analytical domains) |
|  | 20b | Present results of all statistical syntheses conducted. If meta-analysis was done, present for each the summary estimate and its precision (e.g. confidence/credible interval) and measures of statistical heterogeneity. If comparing groups, describe the direction of the effect. | Sections 3.4.1–3.4.5, Sections 3.5.1–3.5.3, and Table 3 (Narrative synthesis of directional associations, multivariable paths, conditional interactions, and contributing study counts $k$) |
|  | 20c | Present results of all investigations of possible causes of heterogeneity among study results. | Sections 3.4–3.5 and Section 4.7 (Narrative contrasts between dedicated ideation scales, proxy screeners, composite risk totals, and repeated-measures designs) |
|  | 20d | Present results of all sensitivity analyses conducted to assess the robustness of the synthesized results. | Section 3.3, Sections 3.4–3.5, and Section 4.7 (Qualitative appraisal of findings across high-quality studies and prospective vs. cross-sectional designs) |
| Reporting biases | 21 | Present assessments of risk of bias due to missing results (arising from reporting biases) for each synthesis assessed. | Section 2.8, Section 3.4, and Section 4.7 (Critical discussion of publication bias, selective reporting, and explicit inclusion of attenuated/null models) |
| Certainty of evidence | 22 | Present assessments of certainty (or confidence) in the body of evidence for each outcome assessed. | Section 3.3, Section 4.7, Section 5, and Supplementary Table S1 (Evidence certainty contextualized via study design limitations, JBI appraisal, and outcome operationalization) |
| **DISCUSSION** | | |  |
| Discussion | 23a | Provide a general interpretation of the results in the context of other evidence. | Section 4 (Introductory text); Subsections 4.1 to 4.5 (Integrative transdiagnostic interpretation across cognitive-affective, emotional, behavioral, interpersonal, and protective domains) |
|  | 23b | Discuss any limitations of the evidence included in the review. | Section 4.7 (Predominance of cross-sectional designs [$85.3\%$], convenience sampling, measurement heterogeneity, self-report reliance, and lack of causal inference) |
|  | 23c | Discuss any limitations of the review processes used. | Section 4.7 (Predefined keyword scope, restriction to peer-reviewed literature in four databases, and absence of prospective registration) |
|  | 23d | Discuss implications of the results for practice, policy, and future research. | Section 4.6 (Practical, campus-level, and clinical assessment implications; multi-tiered intervention targets); Section 4.7 and Section 5 (Research recommendations) |
| **OTHER INFORMATION** | | |  |
| Registration and protocol | 24a | Provide registration information for the review, including register name and registration number, or state that the review was not registered. | Section 2.1 ("The review protocol was not prospectively registered.") |
|  | 24b | Indicate where the review protocol can be accessed, or state that a protocol was not prepared. | Section 2.1 and Section 2.2 (Protocol was not prospectively registered; methods and predefined PECO eligibility criteria are fully detailed in Section 2) |
|  | 24c | Describe and explain any amendments to information provided at registration or in the protocol. | Section 2.1 and Section 2.2 (Not applicable; no prospective registration was undertaken; synthesis structure was established based on empirical outcome characteristics) |
| Support | 25 | Describe sources of financial or non-financial support for the review, and the role of the funders or sponsors in the review. | Funding section (Universidad Nacional de San Agustín de Arequipa, UNSA–INVESTIGA grant PI-08-2025-UNSA); Conflict of interest section (Funder had no role in review conduct or publication decision) |
| Competing interests | 26 | Declare any competing interests of review authors. | Conflict of interest section (Authors declare no commercial or financial conflicts of interest) |
| Availability of data, code and other materials | 27 | Report which of the following are publicly available and where they can be found: template data collection forms; data extracted from included studies; data used for all analyses; analytic code; any other materials used in the review. | Data availability statement and Supplementary Material (Methodological quality appraisal provided in Supplementary Table S1; PRISMA 2020 checklist provided; study data available from corresponding author upon reasonable request) |

*From:*  Page MJ, McKenzie JE, Bossuyt PM, Boutron I, Hoffmann TC, Mulrow CD, et al. The PRISMA 2020 statement: an updated guideline for reporting systematic reviews. BMJ 2021;372:n71. doi: 10.1136/bmj.n71. This work is licensed under CC BY 4.0. To view a copy of this license, visit <https://creativecommons.org/licenses/by/4.0/>
